# The computer, A choreographer? Aesthetic responses to randomly-generated dance choreography by a computer

**DOI:** 10.1016/j.heliyon.2022.e12750

**Published:** 2022-12-30

**Authors:** Kohinoor M. Darda, Emily S. Cross

**Affiliations:** a School of Psychology and Neuroscience, University of Glasgow, UK; b Department of Cognitive Science, Macquarie University, Sydney, Australia; c Penn Center for Neuroaesthetics, University of Pennsylvania, Philadelphia, PA, USA; d MARCS Institute for Brain, Behaviour and Development, Western Sydney University, Sydney, Australia

## Abstract

Is artificial intelligence (AI) changing our culture or creating its own? With advancements in AI and machine learning, artistic creativity is moving to a brave new world of possibility and complexity, while at the same time posing challenging questions, such as what defines something as *art*, what is the role of human creativity in an automated world, and do we evaluate artificial art in the same way as art made by humans? Across two pre-registered and statistically powered experiments we shed light on the nature of aesthetic responses toward computer-generated art by investigating observer prejudices against computer-generated dance choreography, and the impact of expertise and pre-conceived beliefs about the origin of artistic creation. Our results provide substantive evidence that an explicit bias exists among dance experts against computer-generated choreography. The mere belief about a dance work's origin biases aesthetic responses toward artworks among both dance experts and dance naïve participants. The implications of the current study serve to inform several disciplines across the arts and sciences including but not limited to empirical aesthetics, artificial intelligence, engineering, robotics, and social cognition and neuroscience. Along with physical form and content of artificial agents and art productions, the viewers' knowledge and attitudes toward artistic AI and artificial agents will need to be taken into consideration for effective human—computer/human—AI interactions.

**Public Significance Statement**

The current findings suggest that observers have a bias against computer-generated creative productions. The bias is impacted by the observers’ art expertise and preconceived beliefs about the origin of artistic creation. Along with physical form and content of artificial agents and art productions, beliefs and attitudes toward computer-generated art and artificial intelligence will need to be optimised for effective human—computer interactions.

## 1 Introduction

Writers, futurists, scientists, and artists working on or with artificial intelligence (AI) often offer a dystopian view of the future – a world governed by hostile machines that outperform humans. Yet, AI has become an unstoppable force, permeating into our daily lives with autonomous cars and personal AI assistants [1], and surpassing humans in the realm of games such as Go and chess, which were previously thought to be the preserve of human intelligence [2]. Further encroaching into the realm of what many have thought are (or should be) uniquely human pursuits, AI is even producing world-class art [3]. With advancements in AI and machine learning, art and artistic creativity are moving to a brave new world of possibility and complexity, while at the same time posing many challenging questions, such as what defines something as *art*, what is the role of human creativity in an automated world, and is AI changing our culture or creating its own? In the current paper, we aim to shed light on the nature of aesthetic responses toward a particular type of computer-generated art by investigating observer prejudices against computer-generated dance choreography, and the impact of expertise and pre-conceived beliefs about the origin of artistic creation. Answers to these questions have key implications for the future of artistic AI, how we understand the evolving relationship between humans and machines, the role of AI as an impersonator, creator, and collaborator, and ongoing debates about what constitutes the “human” and the “artificial”.

The last few years have seen an exponential growth in “AI artists” – AIVA is an AI that composes movie soundtracks across a wide range of emotions and moods (aiva.ai), dancer Louise Crnkovic-Friis uses a recurrent neural network she calls *chor-nnn,* which is fed 13.5 million spatiotemporal joint positions, producing choreographic sequences very similar to her style [4], and the open source algorithm Generative Pre-Training 2 (GPT-2; [5]) can create poems that can compete with Maya Angelou's [6]. Artist and AI collaborations are on the rise across artistic domains. Computer-generated paintings, music, dance choreography, and poetry are fast establishing themselves as legitimate forms of art, while the line between human and machine is becoming increasingly blurred, especially in the domain of artistic creativity.

Crucial yet understudied questions posed by philosophers, cognitive scientists, psychologists, and artists alike concern whether computer-generated art is evaluated in the same way as art generated by humans, and the extent to which it matters. A few studies that have compared responses to human-generated and computer-generated paintings, music, and poetry suggest that a bias exists against computer-generated and computationally-derived art [[6], [7], [8], [9], [10], [11]].

In a seminal paper by Chamberlain and colleagues [9]; aesthetic responses to computer-generated paintings and human-generated paintings were compared to investigate both implicit and explicit biases against computer-generated paintings. That is, one group of participants rated the paintings first on perceived aesthetic value without any knowledge that some of the paintings were computer-generated, while another group of participants categorised the paintings first into computer-generated and human-generated, and then rated them on perceived aesthetic value. The authors found that images categorised as computer-generated by participants were assigned lower aesthetic value, irrespective of whether they rated or categorised the paintings first. This suggests that both implicit (based on the inherent characteristics of the painting) as well as explicit (when the participant has knowledge about the source of the painting) biases exist against computer-generated paintings [9]. In a similar vein, Moffat and Kelly [7] found that music pieces made by humans were preferred to those made by a computer, but this preference did not change by labelling the pieces of music as made by a human or computer. In contrast, when Kirk and colleagues [8] presented participants with images that were labelled as either taken from an art gallery or made using Photoshop, participants preferred images labelled as originating from the art gallery compared to those images believed to be Photoshopped.

Across previous studies, researchers have also explored how accurately participants can categorise art (or other creative and non-artistic productions) as human- or computer-generated in order to understand whether computers can pass some sort of a “Turing test”. In the domain of empirical aesthetics, participants have generally been able to classify music pieces and paintings as being computer-generated and human-generated, although accuracy was lower for computer-generated art, and higher in those who were art experts [7,9]. In contrast, participants were unable to identify AI-generated poems from poems made by a human, although they preferred human-generated poems to those generated by AI [6]. In a similar vein, in the domain of digital journalism, experiments have investigated people's reactions to algorithmically-generated news articles and those written by journalists [[12], [13], [14]] – participants are generally unable to distinguish between human- and AI-generated texts, yet prefer human-generated texts. These findings suggest that machines can generally pass the Turing test in terms of participants' explicit awareness or detection of an artificial creator. However, the extent to which this also applies to the domain of dance choreography remains to be seem.

Along with accuracy of detection, art expertise also seems to modulate an invididual's aesthetic responses to art more generally. While higher aesthetic ratings for artworks overall is prevalent in previous literature [[15], [16]], whether art expertise modulates aesthetic responses to computer-generated art remains unclear. Chamberlain et al. [9] found no moderation of art interest and education on aesthetic responses to artificial art, whereas findings from other researchers [17] suggest that art educated participants preferred art made by professionals over those labelled as made by children or animals. It is possible that experts show a higher bias against computer-generated art because they are more accurately able to distinguish between computer- and human-generated art.

While previous studies suggest a bias exists against computer-generated art, it is unclear whether this bias is heightened when it is explicit, especially when participants themselves are tasked with labelling the art as human- or computer-generated. An implicit bias would suggest that the inherent qualities of computer-generated art might lead to lower perceived aesthetic value, whereas an explicit bias would suggest a top-down cognitive judgement based on the knowledge about the source of the art. A top-down (explicit) bias may exist when the art presented is computer-generated (and participants are aware that it is computer-generated), but may also exist when the art presented is human-generated but participants *believe* that it is computer-generated. A growing body of social cognition literature suggests that beliefs about humanness (or “artificial-ness”) can profoundly influence social perception [[18], [19], [20]]. These studies present visually identical stimuli in different contexts that vary participants' knowledge or beliefs about the agent's humanness. Thus, any differences in behaviour or responses are independent of the stimuli. No previous study, however, has manipulated the belief about the origin of art. That is, although all art presented may be human generated, it is unclear whether just the belief that the art is computer-generated might bias the observer's aesthetic responses toward the art.

In addition, while much research has focused on participants' perceptions of and biases toward computer-generated paintings, music, and poetry, computer-generated dance choreography has received limited attention, even though choreographers such as Merce Cunningham and Wayne McGregor have integrated AI and dance in their productions for years [21]. As algorithms are being used for developing new movement languages, exploring the role of machines, especially the intangible kind, in choreographing human movement is especially important to advance our understanding of human-machine integration, and a potential role for AI in the dancemaker's toolkit.

One way of investigating people's responses to computer-generated dance choreographies is to present them with choreographies that are generated by a computer, and/or with choreographies that people *believe* are computer-generated (even though they may not be). Along with using artificial intelligence algorithms, one way of conceptualising computer-generated choreographies is by the use of *chance* methods.

Chance methods have been used in creative activities for hundreds of years, and gained prominence in the 19th and 20th centuries [22]. Chance in art can be defined as the intentional use of randomly produced aesthetic elements, where the artist gives up an element of control and assumes only partial responsibility for the artwork. A dose of chance or randomness in art may allow for surprise and trigger a feeling of infinity and totality in the artwork, and lead to innovation and broaden both the artist's and the viewer's thinking [22,23]. Chance methods can vary in how much randomness is incorporated in the artwork. For example, in the domain of music, chance methods can involve a lower degree of chance (e.g., giving a wider-than-usual creative freedom to musicians) or a higher degree of chance (e.g., creating music by tossing coins). An artist may relinquish their control on how chance generates aesthetic elements of an artwork completely, or can continue to decide how these indeterminate parameters are then constructed and applied to make an artwork meaningful [24,25].

Indeed, such chance methods may also include the use of a random number generator implemented in a computing device. Even without using a complex generative process, a random number generator is capable of creating random or *chance*-generated choreographies that are computer-generated by virtue of being implemented in a computing device without any human involvement in the generation of the choreographic sequence. Therefore, in the current study, our computer-generated choreographies reflect randomly generated *chance* choreographies devised by a computer. In this context, no human artists have intervened in the creation of the random movement sequences that constitute the choreography.

### 1.1 Main research questions and hypotheses

In the current study, across two pre-registered and statistically powered experiments, we explore aesthetic responses toward computer-generated dance choreography to answer the following key research questions:

1) Do people exhibit an implicit and/or explicit bias against computer-generated dance choreography (as found previously against computer-generated paintings, poetry, and music)?
2) Is this bias heightened when made explicit, especially when participants themselves label the choreography as computer-generated or human-generated?
3) Does this bias exist when participants *believe* a choreography to be computer-generated (when it is actually human-generated)?
4) Is this bias modulated by dance expertise?
5) Are participants able to accurately categorise choreographies as either computer-generated or human-generated, and are dance experts better at this categorisation?

We predict that both implicit and explicit biases against computer-generated dance choreography will emerge, and these biases will be heightened when explicit, especially when participants themselves categorise the choreographies as human- or computer-generated. We further expect experts to show a stronger bias against computer-generated dance choreography, as well as higher accuracy when categorising choreographies as human-vs. computer-generated. Belief about the source of choreography should also affect aesthetic responses toward choreographies that are believed to be computer-generated even when they are, in reality, human-generated.

## 2 Methods and results

### 2.1 Open science statement

We report how sample size was determined across all experiments, as well as all data exclusions, and all measures used in the study [26,27]. Data pre-processing, statistical analyses, and data visualisations were performed using R (R Core Team, 2018). Following open science initiatives set out by Munafo and colleagues [28], all raw data are available online (https://osf.io/4hsby/). Data analyses for all experiments were preregistered on AsPredicted.org (Experiment 1a and 1b: https://aspredicted.org/ZMS_KYF, Experiment 2: https://aspredicted.org/TLZ_WQ2).

For both Experiments 1 and 2, cumulative link mixed model analyses were executed using the ordinal package (v.2019.12-10) in R v.1.3.1093. (R Core Team). Post-hoc tests were executed using the emmeans package (v.1.5.1). We used an alpha of 0.05 to make inferences, and controlled for multiple comparisons using Tukey-HSD in post-hoc tests. Model fit was compared using the anova () function (Chi-square test).

### 2.2 Stimuli generation

Bharatanatyam is a form of classical Indian dance that features technical abstract dance, storytelling, and a combination of both. Dance movements are performed in a particular posture with the knees flexed, legs bent, and a fixed upper torso, with an emphasis on speed, form, pattern, range, and rhythm. It also has a sophisticated sign language vocabulary based on specific hand gestures and eye movements. For the current study, we used only technical abstract Bharatanatyam choreographies that did not have an interpretative aspect (i.e., without any form of storytelling or enactment). Fifteen dance videos were choreographed by an expert Bharatanatyam dancer. Each dance video was formed of 8 “steps” (or mini-choreographic sequences) of Bharatanatyam Indian classical dance. These formed our human-generated dance choreographies (Mean_duration_ = 9.53 s, SD_duration_ = 0.52 s). To create the computer-generated choreography videos, a total of 120 (15 × 8) different steps were then individually extracted from the intentionally choreographed (human-generated) videos. Each step was then labelled chronologically from 1 to 120. We then used a random number generator to create a sequence of eight randomly generated steps that were joined together to create a sequence. The dancer then learnt this new sequence and recorded the complete sequence. We thus created 15 more choreographic sequences that were not intentionally choreographed by the human choreographer, and were labelled as computer-generated (Mean_duration_ = 9.87 s, SD_duration_ = 0.52 s). The advantage of using Bharatanatyam classical Indian dance was that the randomly generated sequence of movements for the computer-generated choreographies did not technically violate rules of choreography of the dance form. The videos were edited in iMovie with the first 0.5 s of the video faded in, and the last 0.5 s faded out to a black screen. All videos were converted to grayscale and the dancer was instructed to keep a neutral expression throughout for all the videos. All stimuli are available online on the Open Science Framework.

Experiment 1 Implicit and explicit bias against computer-generated choreography.

### 2.3 Sample size justification

We determined the sample size based on a simulation-based power analysis approach using the simr R package [29]. First, we used pilot data (N = 16, 13 females, 8 dance experts, Mean_age_ = 26.50, SD_age_ = 6.06) for beta weight estimation for the following mixed effects model: liking ∼ source of choreography*dance expertise + (1|subject) + (1|item). Second, we simulated data by extending along the sample size, i.e., as a function of different sample sizes. Our focus was the interaction between the source of choreography and dance expertise of participants, and the power analysis suggested that we required a sample size of 100 participants (50 experts and 50 non-experts) with 30 items to have >90% power to detect a significant source of choreography*expertise interaction (more details on the power analyses and the code can be found on the OSF). We therefore aimed to stop data collection when over 100 participants finished the entire survey, with an aim to recruit approximately 50 dance experts and 50 dance-naïve participants.

### 2.4 Participants

Participants were recruited using the online data collection tool Qualtrics. Indian participants were primarily recruited by advertisement on social media and on Prolific (with a filter for those of Indian origin). We intentionally focused our recruitment on an Indian cultural sample as we wanted to recruit both experts and dance naïve participants. Since we assumed that Bharatanatyam experts would primarily be Indian, we did not want any cultural differences between our expert and non-expert participant groups to be confounding any expertise effects that might emerge. Thus, all participants recruited were Indian or of Indian origin. All participants provided informed consent, and reported normal or corrected-to-normal vision. Ethical approval was obtained from the University of Glasgow ethics review board (300200084) and participants were reimbursed with an Amazon gift card of either 6 GBP or Rs. 550 INR.

A total of 166 participants started the online experiment, with 146 participants completing the full experiment. Participants were excluded if they did not pass our attention check questions (see Section 2.2.3; N = 14), had duplicated IP addresses (N = 8), did not provide required demographic information (or entered age below 18; N = 3), or came back to finish the experiment after 24 h (N = 4), or finished the experiment with a duration that was beyond 2SD from the mean time taken by participants to complete the experiment (N = 3). The final sample consisted of 99 participants (74 females, 1 non-binary, and 1 unspecified; Mean_age_ = 28.76, SD_age_ = 9.92) which included 44 experts and 55 dance naïve participants. Out of the 55 dance-naïve participants, 33 were recruited on Prolific (with the country filter set to “Indian”), and the remaining 22 dance naïve and 44 expert participants were recruited via Qualtrics by advertisement on social media.

### 2.5 Tasks and procedure

Participants completed two tasks – a rating task and a categorisation task. In the rating task, participants viewed a dance video on the screen, and were asked to rate it on a 5-point likert scale from low (1) to high (5) with ‘1’ corresponding to ‘not at all’, ‘2’ corresponding to ‘slightly’, ‘3’ corresponding to ‘moderately’, ‘4’ corresponding to ‘very’, and ‘5’ corresponding to ‘extremely’ on the following variables:

1) Familiarity (how familiar is the dance choreography?)
2) Beauty (how beautiful do you find the choreography?)
3) Liking (how much do you like the choreography?)
4) Reproducibility (how reproducible do you think the choreography is?)
5) Enjoyability (how much did you enjoy the choreography?)

We included scales of beauty, liking, and enjoyability as they have been previously used in the domain of empirical aesthetics for both paintings and dance (e.g., [[30], [31], [32]]). Although correlated, they may tap into different components of the cognitive (and aesthetic) reception of a stimulus, and thus may provide a better insight into the aesthetic process as a whole. Further, as previous evidence has suggested that familiarity and reproducibility may influence aesthetic ratings, we included these judgments so as to add them as control variables in our analyses to ensure our results persisted above and beyond the effects of familiarity and reproducibility [[31], [33]].

The order in which these questions were presented was randomized for each item, and the order in which the items (30 dance videos, 15 human-generated choreographies and 15 computer-generated choreographies) were presented was also randomized across participants. Two additional questions appeared randomly during the rating task which served as attention check questions: “how attentive are you while doing this experiment?” and “how honest are you while doing this experiment?” The 5-point likert scale remained the same for the attention check questions as for the other variables. Participants who responded <4 on the 5-point likert scale on the attention check questions were excluded from the analyses.

In the categorisation task, participants categorised the same 30 dance videos they saw during the rating task into either ‘human-generated’ or ‘computer-generated’ depending on whether they thought the source of the dance choreography they watched was of human or computer origin. The order of the items (30 dance videos) was randomized across participants. Approximately half of the participants did the rating task first (“RateFirst”, N = 47, 20 experts), and the remaining participants did the categorisation task first (“CatFirst”, N = 52, 24 experts). This allowed us to test for both an ‘implicit’ and an ‘explicit’ bias. That is, participants who did the rating task first were not explicitly made aware that choreographies could be both computer- or human-generated. Therefore, they assumed all choreographies to be human-generated. Any bias we find in these participants against computer-generated choreography will thus be ‘implicit’ based on the intrinsic qualities of the choreography. In contrast, participants who did the categorisation task first would have ‘explicit’ knowledge during the rating task that some of the choreographies were computer-generated.

In addition to the rating and categorisation task, participants were also asked to answer the following questions with as much detail as possible in order to get qualitative answers exploring the bias for human-generated/against computer-generated choreography:

1) Why did you think a certain choreography was computer-generated? Please explain in as much detail as possible the factors you based your decision on when categorising a certain piece as human-generated or computer-generated.
2) What factors drove your liking for a particular piece of choreography? What factors contributed to you finding a particular piece of choreography beautiful?
3) What do you think about computer-generated choreography? Do you think it is valuable? If yes, why, and if not, why?
4) Are you in support of integrating computer-generated choreography in dance performances, or do you think that computer-generated choreography could mean the end of human creativity?

The experiment started with some demographic questions, and questions that probed participants’ experience with dance (see Supplementary material). Participants then completed the rating and categorisation tasks (with half completing the rating task first, and the other half completing the categorisation task first). The experiment was self-paced, and did not last for more than 60 min for most participants (Mean_duration_ = 49.74 min, SD_duration_ = 29.13 min).

### 2.6 Data analysis

We recorded ratings for each item for each participant on all variables for the rating task. For the categorisation task, we recorded which items were classified as either human-generated or computer-generated by participants (source of choreography as categorised by participants). We also calculated accuracy by calculating the percentage of items that were correctly categorised as computer-generated or human-generated i.e., when the actual source of the dance choreography matched the participant's response.

Experiment 1, therefore, set out to investigate the following specific research questions:

RQ 1.1 *Is there a bias against computer-generated art, and is it heightened for dance experts* (preregistered and confirmatory)*?*
  RQ 1.1a Is the explicit bias stronger than the implicit bias, and is it heightened in dance experts (preregistered and exploratory)*?*
  RQ1.1b Is the bias also present when participants themselves categorise the source of choreography as human-generated or computer-generated (exploratory)?
RQ1.2 Can participants accurately classify choreographies as computer-generated and human-generated? Are experts better at categorisation?

Beauty, liking, and enjoyability ratings were analysed separately. The current analyses differ from our pre-registered analyses in three ways:

1) Our study was powered (>90% power) to detect a source of choreography* dance expertise interaction with N = 100 (50 experts, 50 non-experts). Approximately half of our participants did the rating task first, and the remaining half did the categorisation task first (first task: RateFirst, CatFirst). We therefore include the source of choreography*dance expertise*first task interaction as a fixed effect in the model. We were able to collect N = 99 (44 experts, 55 non-experts). Therefore, while we are powered to detect the source of choreography*dance expertise interaction in the total sample, we are not sufficiently powered to detect the three-way interaction of choreography*dance expertise*first task. Therefore, any findings we report for the three-way interaction are suggestive and exploratory, and not confirmatory.
2) We pre-registered a linear mixed effects analysis using the ‘lme4’ package in R [34]. However, because the data were ordinal in nature, we decided to analyse the ordinal data using cumulative link mixed models by using the ‘ordinal’ package in R [35]. Analysing the data using lme4 yielded similar results.
3) In the preregistered analyses, we included source of choreography (human-generated, computer-generated) and dance expertise (expert, nonexpert) as categorical fixed effects of interest, and the by-subject and by-item intercept as a random factor for the model. However, given recommendations for the “keep it maximal” approach to multilevel modeling [36], we further included the maximal number of random effects that the design permitted for our main effects of interest.

The categorical variables were coded using a deviation coding style where factors sum to zero and the intercept can then be interpreted as the grand mean and the main effects can be interpreted similarly to a conventional ANOVA (http://talklab.psy.gla.ac.uk/tvw/catpred/). As such, the categorical variables of source of choreography, dance expertise, and first task were coded as 0.5 (human-generated/expert/CatFirst) and −0.5 (computer-generated/nonexpert/RateFirst). An ordinal logistic regression was employed in the form of a cumulative-link mixed model (*ordinal* package, “clmm” function [35], using logit (log-odds) as link, and flexible thresholds between the ordinal scores. We chose this approach because the dependent or outcome variables ‘beauty’, ‘liking’, and ‘enjoyability’ ratings were ordinal in nature (ratings on a Likert scale 1–5). The model thus measures the probability of specific ratings being above certain thresholds without the assumption that the thresholds are symmetric or equidistant from each other.

In order to address RQ1.1. (i.e., whether there is a bias against computer-generated art, and whether this bias is stronger in dance experts), across all participants (N = 99), we included the two-way interaction of source of choreography and dance expertise as a fixed effect in the model. For random effects, we included the maximal number of random effects that the design permitted. The complexity of the random structure was reduced if the results showed failure in model convergence or a singular fit. The final model used was:

clmm(beauty/liking/enjoyability ∼ 1 + source of choreography*dance expertise + (1 + source of choreography | *subject) + (1 + dance expertise* | *item), link = “logit”, threshold = “flexible”)*

Next, to address RQ1.1a i.e. whether a bias against computer-generated art is heightened in dance experts, and is stronger when the bias is explicit, we constructed a more complex model, including the three way interaction of source of choreography (human-generated, computer-generated), dance expertise (expert, nonexpert), and first task (RateFirst, CatFirst) as a fixed effect in the model. For random effects, we included the maximal number of random effects that the design permitted. The complexity of the random structure was reduced if the results showed failure in model convergence or a singular fit. The final model used was:

clmm(beauty/liking/enjoyability ∼ 1 + source of choreography*dance expertise*first task + (1 + source of choreography | subject) + (1 + dance expertise * first task | *item), link = “logit”, threshold = “flexible”)*

For both the analyses above, to test whether source of choreography, dance expertise, and first task modulated beauty, liking, and enjoyability ratings above and beyond the subjective factors that participants rated the paintings on (familiarity and reproducibility), as well as how they were recruited (via Prolific or social media) we further added the subjective variables of familiarity and reproducibility, and recruitment platform (Prolific, Social Media) as fixed effects to the model. Thus, the final model used was:

clmm(beauty/liking ∼ 1 + source of choreography*dance expertise*first task + familiarity + reproducibility + recruitment_platform + (1 + source of choreography | subject) + (1 + dance expertise * first task | *item), link = “logit”, threshold = “flexible”)*

In all the analyses above, the factor ‘source of choreography’ was coded according to whether the choreography was actually computer- or human-generated (see section 2.2). As mentioned previously, participants who did the rating task first were not explicitly made aware that the choreographies were human- or computer-generated. If a bias does exist in this case, a preference for human-generated choreographies over computer-generated ones might be reported irrespective of whether participants can accurately identify the choreography as human- or computer-generated, and without explicit knowledge of the fact that choreographies could in fact be human- or computer-generated. In this case, an implicit bias against computer-generated choreography would indicate that certain intrinsic qualities are associated with the computer-generated choreographies that participants do not like, enjoy, or find beautiful compared to human-generated choreographies. In contrast, a bias against computer-generated choreography may only arise or be heightened in participants who did the categorisation task first as they were explicitly made aware before the rating task that the choreographies could be computer- or human-generated. Therefore, a bias against computer-generated choreography in this case would suggest an explicit bias as participants have explicit knowledge about the source of the choreography.

Similarly, a bias may arise or be heightened only when participants themselves categorise the choreography as human- or computer-generated (irrespective of whether the choreography is actually human- or computer-generated). This may be the case especially in dance naïve participants who may not be able to accurately identify the difference in the source of choreography (whether implicit or explicit). Thus, to test RQ1.1b, we repeated the mixed effects model analyses with the factor ‘source of choreography - ppt’ coded as ‘human-generated or ‘computer-generated’ as categorised by the participants in the categorisation task.

Finally, to address RQ 1.2. i.e., to explore participants’ accuracy in categorising choreographies as human-generated or computer-generated, we subjected accuracy scores to a 2 (source: computer-generated, human-generated) x 2 (expertise: dance experts, non-experts) factorial analysis of variance (ANOVA). The main research questions and cumulative link mixed effects models used in Experiment 1 and 2 are summarised in Table 1.

**Table 1:** Research questions and corresponding analyses performed.

Number	Research Question and hypotheses	Model/ANALYSES Used
Experiment 1		
RQ1.1.	*Is there a bias against computer-generated art, and is it heightened for dance experts?*	clmm(beauty/liking/enjoyability ∼1 + source of choreography*dance expertise + (1 + source of choreography | *subject) + (1 + dance expertise* | *item), link = “logit”, threshold = “flexible”)*
	H: A bias exists against computer-generated dance choreography, and the bias is heightened for experts	
RQ1.1a.	*Is the explicit bias stronger than the implicit bias, and is it heightened in dance experts?*	clmm(beauty/liking/enjoyability ∼1 + source of choreography*dance expertise*first task + (1 + source of choreography | subject) + (1 + dance expertise * first task | *item), link = “logit”, threshold = “flexible”)*
	H: An explicit bias against computer-generated dance choreography is stronger than an implicit bias, and the bias is heightened for experts	
RQ1.1b.	*Is the bias also present when participants themselves categorise the source of choreography as human-generated or computer-generated?*	clmm(beauty/liking/enjoyability ∼1 + source of choreography (participants)*dance expertise*first task + (1 + source of choreography (participants) | subject) + (1 + dance expertise * first task | *item), link = “logit”, threshold = “flexible”)*
	H: The bias against computer-generated dance choreography is present when the source of choreography is categorised by the participants themselves	
RQ1.2.	*Can participants accurately classify choreographies as computer-generated and human-generated? Are experts better at categorisation?*	2 (source: computer-generated, human-generated) x 2 (expertise: dance experts, non-experts) factorial analysis of variance (ANOVA)
	H: Dance experts are more accurate when classifying choreographies as computer- or human-generated than non-experts	
Experiment 2		
RQ2	*Is there a bias against dance choreography when it is****believed****to be computer-generated, and is this bias heightened for dance experts?*	clmm(beauty/liking ∼1 + source of choreography*dance expertise + (1 + source of choreography | *subject) + (1 + dance expertise* | *item), link = “logit”, threshold = “flexible”)*
	A bias exists against choreographies that are believed to be computer-generated (even when they may not be), and this bias is higher in experts	

### 2.7 Results – Experiment 1

#### 2.7.1 Categorisation task

**Fig. 1:**
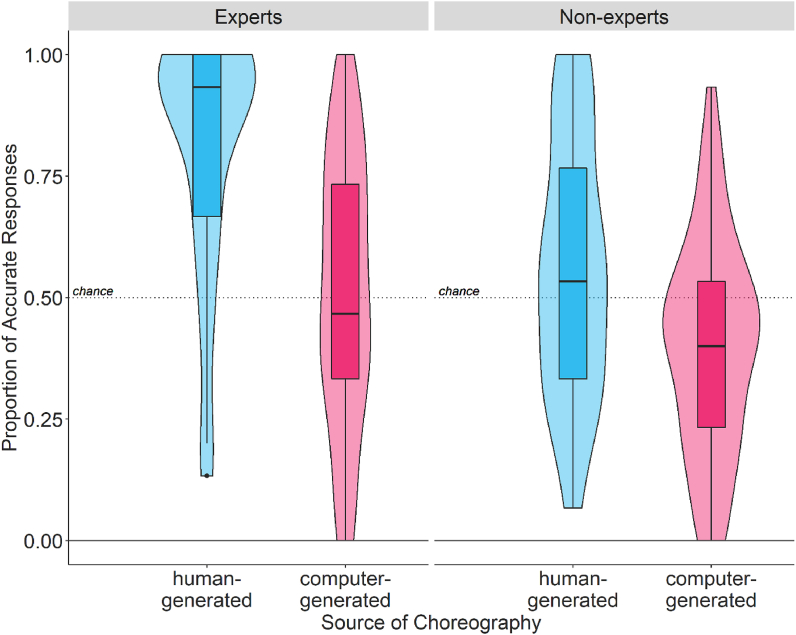
Proportion of accurate responses for experts and non-experts for human- and computer-generated choreography. Dashed line represents 50% accuracy (or chance).

Accuracy on the categorisation task across source of choreography, expertise, and first task are provided in Table S1. A 2 (source of choreography: human-generated, computer-generated) × 2 (dance expertise: experts, nonexperts) × 2 (first task: RateFirst, CatFirst) ANOVA showed a significant main effect of expertise (F (1,190) = 25.13, p < .001, pes = 0.115), a significant main effect of source of choreography (F (1,190) = 39.10, p < .001, pes = 0.16), and a significant dance expertise*source of choreography interaction (F (1,190) = 3.76, p = .05, pes = 0.02). Posthoc tests suggest that experts are more accurate than non-experts (*EMM* = 0.18, *SE* = 0.04, t (190) = 5.01, p < .001). Accuracy for human-generated choreography is higher than computer-generated choreography (*EMM* = 0.23, *SE* = 0.36, t (190) = 6.24, p < .001) both for experts (*EMM* = 0.30, *SE* = 0.05, t (190) = 5.48, p < .001) as well as for nonexperts (*EMM* = 0.16, *SE* = 0.05, t (190) = 3.24, p = .001), with the difference being higher for experts compared to non-experts (*EMM* = 0.14, *SE* = 0.07, t (190) = 1.90, p = .05; see Fig. 1). The main effect of first task, and other two-way and three-way interactions were not significant (see Table S2).

#### 2.7.2 Rating task

Mean ratings for familiarity, reproducibility, beauty, liking, and enjoyability across source of choreography, expertise, and first task are provided in Supplementary Table S3 and Figures S1 and S2.

RQ1.1: Is there a bias against computer-generated art, and is it heightened for dance experts (preregistered and confirmatory)?

Main model = clmm(beauty/liking/enjoyability ∼ 1 + source of choreography*dance expertise + (1 + source of choreography | subject) + (1 + dance expertise | item), link = “logit”, threshold = “flexible”)

The results of a cumulative link mixed effects model for beauty, liking, and enjoyability ratings showed that dance expertise (beauty: *β* = 1.00, p = .01; liking: *β* = 1.04, p = .006; enjoyability: *β* = 0.96, p = .01), source of choreography (beauty: *β* = 0.64, p = .015; liking: *β* = 0.67, p = .010; enjoyability: *β* = 0.71, p = .01), and the two way interactions of dance expertise by source of choreography (beauty: *β* = 1.85, p < .001; liking: *β* = 1.50, p < .001; enjoyability: *β* = 1.38, p < .001) had an effect on the ratings of beauty, liking, and enjoyability. In order to test whether the effect of source of choreography and dance expertise affected our dependent variables irrespective of the recruitment platform used (Prolific or social media), and above and beyond the subjective variables on which participants rated the dance choreography (familiarity, reproducibility), we ran an additional model including recruitment platform as a categorical fixed effect (coded 0.5 for “Prolific” and −0.5 for “Social Media”), and familiarity and reproducibility as continuous fixed effects in the model.

Subj model = clmm(beauty/liking/enjoyability ∼ 1 + source of choreography*dance expertise + recruitment_platform + familiarity + reproducibility + (1 + source of choreography | *subject) + (1 + dance expertise* | *item), link = “logit”, threshold = “flexible”)*

**Fig. 2:**
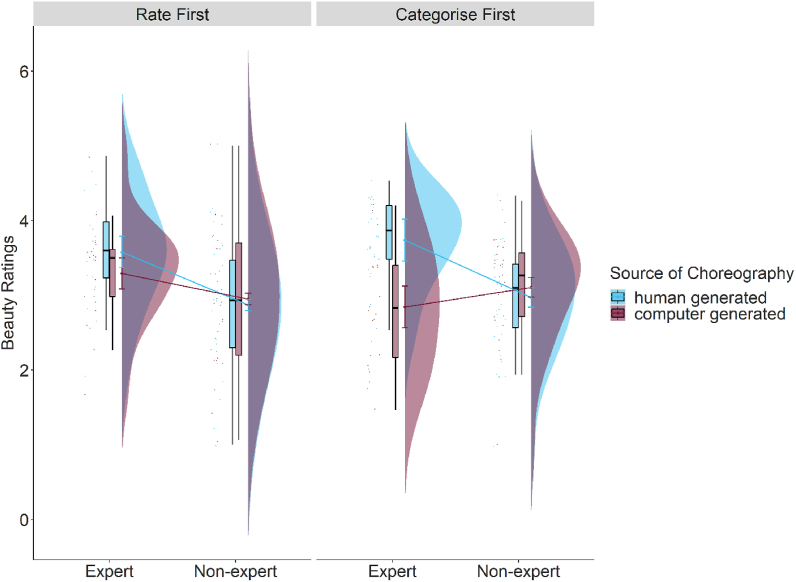
The effect of dance expertise and source of choreography on beauty ratings for participants who did the rating task first (implicit bias) and participants who did the categorisation task first (explicit bias). Results for liking and enjoyability ratings are similar (in the same direction, see Figures S3 and S4, Tables S4–S7). Data is visualised using raincloud plots [2].

The results showed that recruitment platform (beauty: β = −1.30, p = .005; liking: β = −0.82, p = .06; enjoyability: *β* = −0.76, p = .08), familiarity (beauty: β = 0.58, p < .001; liking: β = 0.61, p < .001; enjoyability: *β* = 0.61, p < .001), reproducibility (beauty: *β* = 0.60, p < .001; liking: β = 0.58, p < .001; enjoyability: β = 0.60, p < .001), source of choreography (beauty: β = 0.49, p = .039; liking: β = 0.49, p = .04; enjoyability: *β* = 0.53, p = .03), and the two way interactions of dance expertise by source of choreography (beauty: β = 1.36, p < .001; liking: β = 0.95, p = .008; enjoyability: *β* = 0.83, p = .02) had an effect on the ratings of beauty, liking, and enjoyability. The model including recruitment platform, familiarity, and reproducibility significantly improved the model without these variables added as fixed effects (beauty: AIC_main_ = 7107.21, AIC_subj_ = 6841.11, p < .001; liking: AIC_main_ = 7245.22, AIC_subj_ = 6980.32, p < .001; enjoyability: AIC_main_ = 7235.58, AIC_subj_ = 6961.30, p < .001). Importantly, our main effect of interest (i.e., the two-way interaction between dance expertise and source of choreography) still predicted ratings of beauty, liking, and enjoyability, irrespective of the effects of familiarity, reproducibility, and recruitment platform (see Fig. 2, Tables S4 and S5).

As predicted, a bias against computer-generated dance choreography was found, but only amongst dance experts. Specifically, experts showed lower ratings of beauty, liking, and enjoyability for computer-generated dance choreography (beauty: *M* = −0.32 [−1.09, 0.46], *SE* = 0.23; liking: *M* = −0.17 [−0.93, 0.58], *SE* = 0.39; enjoyability: *M* = −0.13 [−0.91, 0.64], *SE* = 0.39) compared to human-generated choreography (beauty: *M* = 0.85 [0.05, 1.65], *SE* = 0.41, p < .001; liking: *M* = 0.79 [0.02, 1.57], *SE* = 0.39, p = .001; enjoyability: *M* = 0.81 [0.03, 1.60], *SE* = 0.40) whereas non-experts did not show a difference between computer-generated (beauty: *M* = 0.15 [−0.35, 0.66], *SE* = 0.31; liking: *M* = −0.18 [−0.68, 0.32], *SE* = 0.25; enjoyability: *M* = −0.17 [−0.68, 0.33], *SE* = 0.26) and human-generated choreographies (beauty: *M* = −0.03 [−0.56, 0.49], *SE* = 0.27, p = .41; liking: *M* = −0.18, *SE* = 0.25, p = .78; enjoyability: *M* = −0.05 [−0.56, 0.45], *SE* = 0.26; see Fig. 2).

RQ1.1a: Is the explicit bias stronger than the implicit bias, and is it heightened in dance experts (preregistered and exploratory)?

In order to test whether the explicit bias is stronger than the implicit bias we ran the following model:

Max model = clmm(beauty/liking/enjoyability ∼ 1 + source of choreography*dance expertise*first task + (1 + source of choreography | subject) + (1 + dance expertise * first task | *item), link = “logit”, threshold = “flexible”)*

The results showed a main effect of dance expertise (beauty: *β* = 1.02, p = .01; liking: *β* = 1.06, p = .004; enjoyability: β = 0.98, p = .01), source of choreography (beauty: β = 0.60, p = .02; liking: β = 0.63, p = .01; enjoyability: β = 0.68, p = .01), a two way interaction between dance expertise and source of choreography (beauty: β = 1.78, p < .001; liking: β = 1.41, p < .001; enjoyability: β = 1.32, p < .001), and between source of choreography and first task (beauty: β = 0.69, p = .03; liking: β = 0.77, p = .02; enjoyability: β = 0.64, p = .05), and a three way interaction between first task, source of choreography, and dance expertise (beauty: β = 1.65, p = .01; liking: β = 2.17, p = .001; enjoyability: β = 1.72, p = .01). No other main effects or two-way interactions were significant (see Table S6). To test whether the effect of source of choreography, dance expertise, and first task affected our dependent variables irrespective of the recruitment platform used (Prolific or social media), and above and beyond the subjective variables on which participants rated the dance choreography (familiarity, reproducibility), we ran an additional model including recruitment platform as a categorical fixed effect and familiarity and reproducibility as continuous fixed effects in the model.

Max subj model = clmm(beauty/liking ∼ 1 + source of choreography*dance expertise*first task + familiarity + reproducibility + recruitment_platform + (1 + source of choreography | subject) + (1 + dance expertise * first task | *item), link = “logit”, threshold = “flexible”)*

**Fig. 3:**
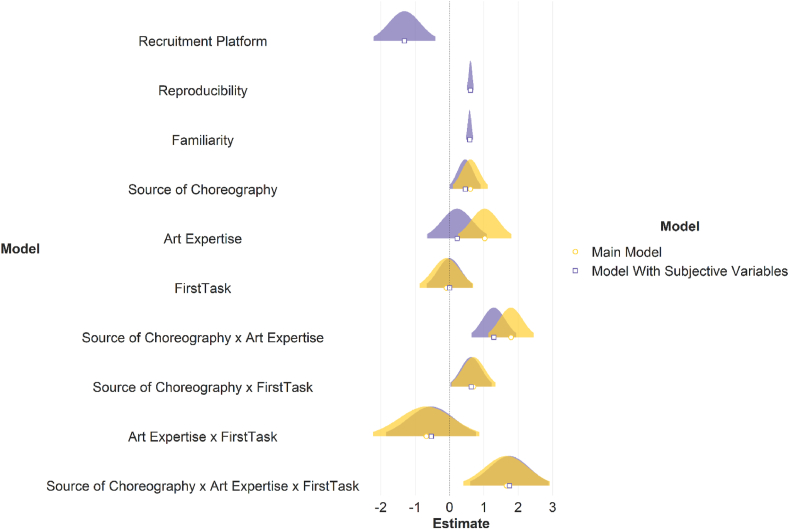
Model comparison for beauty ratings (results for liking and enjoyability were in a similar direction). For the outcome variable of beauty ratings, beta estimates for the model including the three way interaction between dance expertise, source of choreography, and first task (in yellow; light grey if printed in black and white) and the model including the three way interaction as well as the subjective variables of familiarity and reproducibility, and the variable of recruitment platform (in purple; dark grey if printed in black and white) are plotted for each predictor variable along with their corresponding uncertainties (95% confidence interval width for a normal distribution for each estimate). Distributions are rescaled to match the height of each distribution. (For interpretation of the references to colour in this figure legend, the reader is referred to the Web version of this article.)

Results showed that the main effects of source of choreography (beauty: β = 0.46, p = .05; liking: β = 0.45, p = .05; enjoyability: β = 0.50, p = .04), familiarity (beauty: β = 0.58, p < .001; liking: β = 0.62, p < .001; enjoyability: β = 0.62, p < .001), reproducibility (beauty: β = 0.61, p < .001; liking: β = 0.59, p < .001; enjoyability: β = 0.61, p < .001), recruitment platform (beauty: β = −1.31, p = .06; liking: β = −0.82, p = .006; enjoyability: β = −0.78, p = .08), the two way interaction between dance expertise and source of choreography (beauty: β = 1.29, p < .001; liking: β = 0.86, p = .01; enjoyability: β = 0.76, p = .03), and between source of choreography and first task (beauty: β = 0.63, p = .04; liking: β = 0.69, p = .02; enjoyability: β = 0.58, p = .06), as well as the three way interaction between first task, source of choreography, and dance expertise (beauty: β = 1.74, p = .002; liking: β = 2.23, p < .001; enjoyability: β = 1.81, p = .003) predicted ratings of beauty, liking, and enjoyability. No other main effects and interactions were significant (see Table S7). The model including recruitment platform, familiarity, and reproducibility significantly improved the model without these variables added as fixed effects (beauty: AIC_main_ = 7115.42, AIC_subj_ = 6846.84, p < .001; liking: AIC_main_ = 7249.91, AIC_subj_ = 6979.65, p < .001; enjoyability: AIC_main_ = 7240.44, AIC_subj_ = 6964.41, p < .001). Importantly, our main effect of interest i.e. the three-way interaction between first task, dance expertise and source of choreography still predicted ratings of beauty, liking, and enjoyability, above and beyond our *max model* (see Figs. 2 and 3, Table S7). To further probe our three way interaction, we computed the difference between human generated choreography and computer generated choreography for experts and non-experts, separately for participants who did the rating task first, and participants who did the categorisation task first. Posthoc analyses (corrected for multiple comparisons using Tukey's HSD) suggested that ratings for human-generated choreographies were higher than computer-generated choreographies among dance experts but only when they did the categorisation task first (beauty: *estimate* = −1.85, *SE* = 0.41, 95% *CI* [−2.90, −0.80], p < .001; liking: *estimate* = −1.79, *SE* = 0.39, 95% *CI* [−2.80, −0.79], p < .001; enjoyability: *estimate* = −1.63, *SE* = 0.41, 95% *CI* [−2.68, −0.57], p < .001).

RQ1.1b: Is the bias also present when participants themselves categorise the source of choreography as human-generated or computer-generated (exploratory)?

In the analyses above, the factor ‘source of choreography’ was coded according to whether the choreography was actually computer- or human-generated (i.e., as coded by the experimenter). To test whether the bias is also present or is heightened when participants themselves categorise the choreography as human- or computer-generated, we repeated the analysis above (*Max subj model*) with the fixed effect of source of choreography as categorised by the participant. The maximal model which included varying interaction term of dance expertise and first task for each item did not converge, and so we included *(1 + source of choreography_ppt* | *subject)* and *(1 + dance expertise* | *item)* as separate random slopes and intercepts.

Max subj model_ppt = clmm(beauty/liking/enjoyability ∼ 1 + source of choreography_ppt*dance expertise*first task + familiarity + reproducibility + recruitment_platform + (1 + source of choreography_ppt | *subject) + (1 + dance expertise* | *item) + (1 + first task* | *item), link = “logit”, threshold = “flexible”)*

**Fig. 4:**
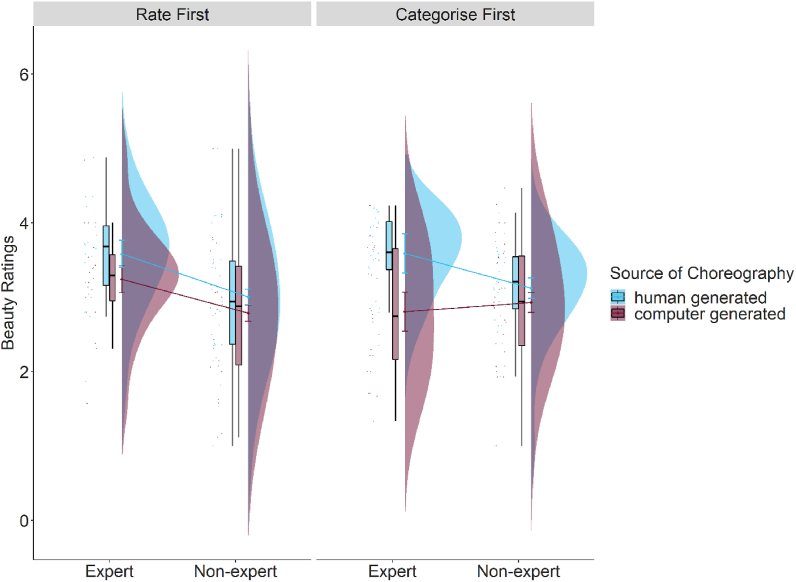
The effect of dance expertise and source of choreography as categorised by participants on beauty ratings for participants who did the rating task first (implicit bias) and participants who did the categorisation task first (explicit bias). Results for liking and enjoyability ratings are similar (in the same direction, see Table S8).

Results showed that the main effects of source of choreography as categorised by participants (beauty: β = 0.62, p < .001; liking: β = 0.46, p = .001; enjoyability: β = 0.42, p = .002, familiarity (beauty: β = 0.62, p < .001; liking: β = 0.67, p < .001; enjoyability: β = 0.65, p < .001), reproducibility (beauty: β = 0.60, p < .001; liking: β = 0.57, p < .001; enjoyability: β = 0.57, p < .001), recruitment platform (beauty: −1.16, p = .01) predicted ratings of beauty, liking, and enjoyability. The two way interaction of source of choreography as categorised by participants and first task (beauty: 0.45, p = .09; liking: β = 0.58, p = .04; enjoyability: β = 0.57, p = .03) and the three way interaction of dance expertise, source of choreography as categorised by participants, and first task (beauty: 0.95, p = .07; liking: β = 1.11, p = .05; enjoyability: β = 1.00, p = .06) predicted ratings of beauty, liking and enjoyability. To further investigate our three-way interaction, we tested the difference between ratings for human- and computer-generated choreographies across our levels of expertise and first task. Similar to when the source of choreography was classified by the experimenter, we found a difference in ratings between human- and computer-generated choreographies only for dance experts who did the categorisation task first (beauty: *estimate* = −1.26, *SE* = 0.27, 95% *CI* [−1.96, −0.56], p < .001; liking: *estimate* = −1.18, *SE* = 0.29, 95% *CI* [−1.92, −0.44], p < .001; enjoyability: *estimate* = −1.03, *SE* = 0.27, 95% *CI* [−1.73, −0.33], p < .001; see Fig. 4, Table S8).

### 2.8 Discussion for Experiment 1

The results from Experiment 1 suggest that, as hypothesized, participants showed a bias against computer-generated dance choreography. However, the bias was present only among dance experts, and was explicit. That is, the bias existed only when dance experts were aware that some choreographies are computer-generated. This explicit bias persisted (and was heightened) in dance experts when they themselves categorised the choreographies as computer-vs. human-generated. Further, experts were better able to categorise both human- and computer-generated choreographies than non-experts.

In Experiment 2, we investigate the extent to which such a bias against computer-generated dance choreography persists when participants *believe* that a choreography is computer-generated.

Experiment 2 The impact of belief about the source of choreography on aesthetic responses to dance choreography.

### 2.9 Sample size justification

Like Experiment 1, we determined the sample size based on a simulation-based power analysis approach using the simr R package [29]. First, we used pilot data (N = 13, 9 females, 6 dance experts, Mean_age_ = 28.23, SD_age_ = 8.72) for beta weight estimation for the following mixed effects model: liking ∼ belief of choreography*dance expertise + (1|subject) + (1|item). Second, we simulated data by extending along the sample size, i.e., as a function of different sample sizes. Our focus was the interaction between the source of choreography and dance expertise of participants, and the power analysis suggested that we required a sample size of 80 participants (40 experts and 40 non-experts) with 15 items to have >80% power to detect a significant source of choreography*expertise interaction (more details on the power analyses and the code can be found on the OSF). We therefore aimed to stop data collection when over 80 participants finished the entire survey, with an aim to recruit approximately 40 dance experts and 40 dance-naïve participants.

### 2.10 Participants

The survey was designed using the online data collection tool Qualtrics. Indian participants were primarily recruited by advertisement on social media and on Prolific (with a filter for those of Indian origin). Again, like Experiment 1, we intentionally focused our recruitment on an Indian cultural sample as we wanted to recruit both experts and dance naïve participants. All participants provided informed consent, and reported normal or corrected-to-normal vision. The experiment lasted not more than 30 min, and participants were reimbursed with an Amazon gift card of either 3 GBP or Rs. 250 INR, or paid 3GBP on Prolific.

A total of 118 participants started the online experiment, with 103 participants completing the full experiment. Participants were excluded if they had duplicated IP addresses (N = 11), did not provide required demographic information (or entered age below 18; N = 5), did not do the experiment in one sitting (N = 3) or finished the experiment with a duration that was beyond 2SD from the mean time taken by participants to complete the experiment (N = 3). The final sample consisted of 81 participants (61 females; Mean_age_ = 27.39, SD_age_ = 7.04) which included 43 experts and 38 dance naïve participants. Out of the 38 dance-naïve participants, 25 were recruited on Prolific (with the country filter set to “Indian”), and 13 were recruited on social media. All 43 dance experts were recruited with the help of social media.

### 2.11 Tasks and procedure

Participants completed a rating task in which they viewed a dance video on the screen, and were asked to rate it on a 5-point likert scale from low (1) to high (5) with ‘1’ corresponding to ‘not at all’, ‘2’ corresponding to ‘slightly’, ‘3’ corresponding to ‘moderately’, ‘4’ corresponding to ‘very’, and ‘5’ corresponding to ‘extremely’ on the following variables:

1) Beauty (how beautiful do you find the choreography?)
2) Liking (how much do you like the choreography?)

The order in which these questions were presented was randomized for each item, and the order in which the items (15 human-generated choreographies) were presented was also randomized across participants. Before doing the rating task, in order to manipulate the belief of participants about the source of the choreography (computer- or human-generated), participants were shown an elaborate video (cover story) about how computer-generated choreographies were made. The video is available on OSF (https://osf.io/4hsby/) and was made in such a way that participants would believe that some of the choreographies they later saw were computer-generated (made by an AI algorithm) and some were human-generated, although all choreographies presented were human-generated. For approximately half of the participants (N = 35, 22 experts), half the videos (items 1–7) were preceded by the statement “this choreography is computer-generated”, and the remaining half (items 8–15) were preceded by the statement “this choreography is human-generated”. For the remaining participants (N = 46, 21 experts), items 1–7 were preceded by the statement “this choreography is human-generated” and items 8–15 were preceded by the statement “this choreography is computer-generated.”

In addition to the rating task, participants were also asked to answer the same questions as Experiments 1a and 1b with as much detail as possible in order to get qualitative answers exploring the bias for human-generated/against computer-generated choreography, with one additional question to test whether participants believed our cover story:

1) All pieces of choreography you saw in this survey were actually human-generated. No computer algorithm was used for the choreography. However, we made you believe that certain pieces of choreography were computer-generated. Did you notice this manipulation?

The experiment started with some demographic questions, and questions that probed participants’ experience with dance (see Supplementary material). Participants then watched our belief manipulation/cover story video, and then completed the rating task, followed by the qualitative questions. The experiment was self-paced, and did not last for more than 30 min for most participants (Mean_duration_ = 28.81 min, SD_duration_ = 18.85 min).

### 2.12 Data analysis

We recorded ratings for each item for each participant on all variables for the rating task. Experiment 2 set out to investigate the following specific research question:

*RQ 2: Is there a bias against dance choreography when it is****believed****to be computer-generated, and is this bias heightened for dance experts* (preregistered and confirmatory)*?*

Beauty and liking ratings were analysed separately. The current analyses differ from our pre-registered analyses in two ways:

1) Our study was powered (>80% power) to detect a source of choreography* dance expertise interaction with N = 80 (40 experts, 40 non-experts). We pre-registered a linear mixed effects analysis using the ‘lme4’ package in R [34]. However, because the data were ordinal in nature, we decided to analyse the ordinal data using cumulative link mixed models by using the ‘ordinal’ package in R [35]. Analysing the data using lme4 yielded similar results.
2) In the preregistered analyses, we included source of choreography (human-generated, computer-generated) and dance expertise (expert, nonexpert) as categorical fixed effects of interest, and the by-subject and by-item intercept as a random factor for the model. However, given recommendations for the “keep it maximal” approach to multilevel modeling [36], we further included the maximal number of random effects that the design permitted.

The categorical variables were coded using a deviation coding style where factors sum to zero and the intercept can then be interpreted as the grand mean and the main effects can be interpreted similarly to a conventional ANOVA (http://talklab.psy.gla.ac.uk/tvw/catpred/). As such, the categorical variables of source of choreography and dance expertise were coded as 0.5 (human-generated/expert) and −0.5 (computer-generated/nonexpert). An ordinal logistic regression was employed in the form of a cumulative-link mixed model (*ordinal* package, “clmm” function [35], using logit (log-odds) as link, and flexible thresholds between the ordinal scores. We chose this approach because the dependent or outcome variables ‘beauty’ and ‘liking’ ratings were ordinal in nature (ratings on a Likert scale 1–5). The model thus measures the probability of specific ratings being above certain thresholds without the assumption that the thresholds are symmetric or equidistant from each other.

In order to address RQ2. i.e. whether there is a bias against dance choreography when it is believed to be computer-generated, and whether this bias is stronger in dance experts, across all participants (N = 81), we included the two-way interaction of source of choreography and dance expertise as a fixed effect in the model. For random effects, we included the maximal number of random effects that the design permitted. The complexity of the random structure was reduced if the results showed failure in model convergence or a singular fit. The final model used was:

clmm(beauty/liking ∼1 + source of choreography*dance expertise + (1 + source of choreography | subject) + (1 + dance expertise | item), link = “logit”, threshold = “flexible”)

To check if participants believed our cover story, we read the answers provided by participants on the question about whether they noticed our belief manipulation. We further excluded the participants who did not believe our cover story and ran the same analysis as above.

### 2.13 Results – Experiment 2

*RQ 2: Is there a bias against dance choreography when it is****believed****to be computer-generated, and is this bias heightened for dance experts* (preregistered and confirmatory)*?*

Main model = clmm(beauty/liking ∼ 1 + source of choreography*dance expertise + (1 + source of choreography | *subject) + (1 + dance expertise* | *item), link = “logit”, threshold = “flexible”)*

**Fig. 5:**
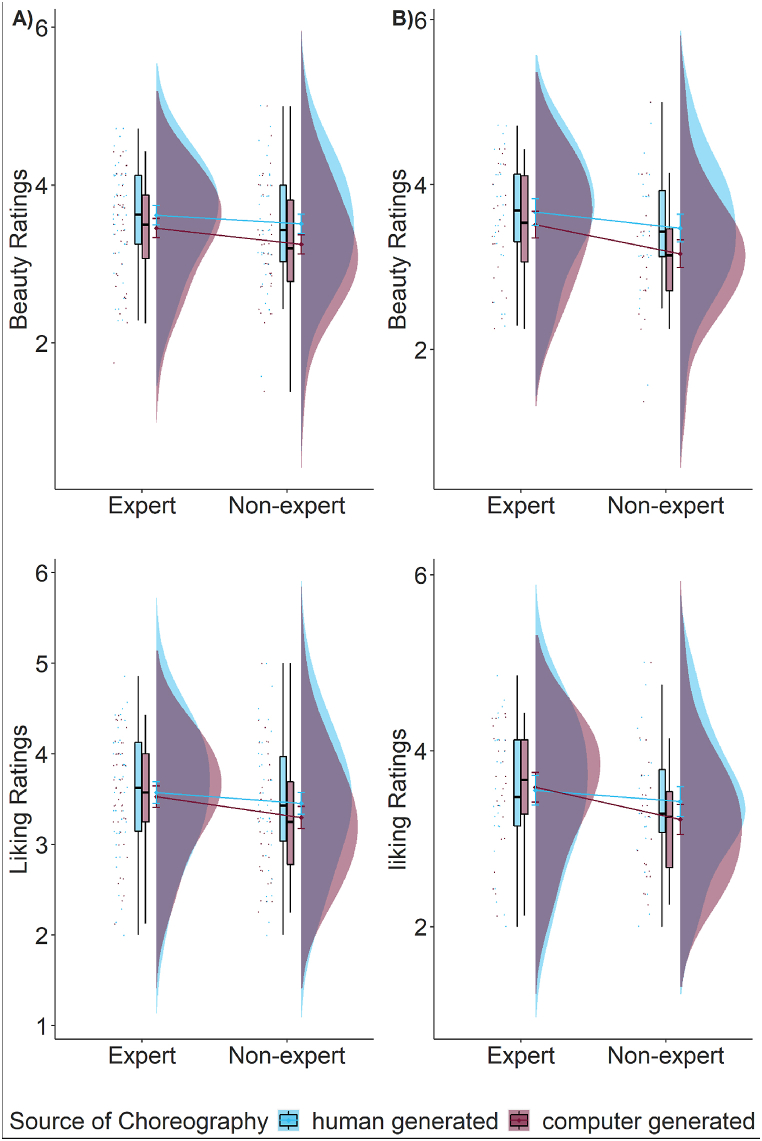
Experiment 2. The effect of source of choreography on beauty and liking ratings for all participants (A) and excluding participants who did not fully believe our cover story (B).

Results showed that the fixed effect of source of choreography predicted ratings of beauty and liking (beauty: *β* = 0.29, p = .04; liking: *β* = 0.29, p = .04). The main effect of dance expertise and the two-way interaction of source of choreography and dance expertise were not significant (see Table S9, Fig. 5A). To ensure that the effect of source of choreography persisted irrespective of whether participants were recruited on social media or on Prolific, we further tested the model including recruitment platform as a fixed effect in the model. The final model tested was:

Subj model = clmm(beauty/liking ∼ 1 + source of choreography*dance expertise + recruitment_platform + (1 + source of choreography | *subject) + (1 + dance expertise* | *item), link = “logit”, threshold = “flexible”)*

Adding the main effect of recruitment platform did not significantly improve the model (beauty: AIC_main_ = 3080.67, AIC_subj_ = 3082.58, p = .75; liking: AIC_main_ = 3064.18, AIC_subj_ = 3065.97, p = .63). Source of choreography continued to predict ratings of beauty and liking even when recruitment platform was included in the model (beauty: *β* = 0.29, p = .04; liking: *β* = 0.29, p = .04). The effects of dance expertise and the two-way interaction between dance expertise and source of choreography were not significant (see Table S10). As predicted, participants showed higher ratings of beauty and liking for human-generated choreographies (beauty: M = 1.36 [0.82, 1.89], SE = 0.27; liking: M = 1.27 [0.71, 1.82], SE = 0.28) compared to computer-generated choreographies (beauty: M = 0.79 [0.27, 1.32], SE = 0.27, p < .001; liking: M = 0.97 [0.45, 1.50], SE = 0.27, p = .04).

We repeated these analyses for participants who responded that they believed our cover story (N = 49, 26 experts; participants who explicitly said “yes” (yes = they believed our cover story) were included, participants who were unsure or said “no” (no = they did not believe our cover story) were excluded from the analyses). Results were similar such that the main effect of source of choreography predicted beauty ratings (but not liking ratings; beauty: *β* = 0.61, p < .001; liking: *β* = 0.24, p = .24) irrespective of the recruitment platform (see Tables S9 and S10). Participants showed higher ratings of beauty but not liking for human-generated choreographies (beauty: M = 1.51 [0.81, 2.20], SE = 0.36; liking: M = 1.32 [0.64, 2.00], SE = 0.35) compared to computer-generated choreographies (beauty: M = 0.90 [0.22, 1.57], SE = 0.34, p < .001; liking: M = 1.08 [0.47, 1.70], SE = 0.31, p = .24; see Fig. 5A and B).

### 2.14 Discussion for Experiment 2

Findings from Experiment 2 suggest that a pre-conceived belief about the origin of artistic creation also influenced aesthetic ratings. Participants, both dance experts and dance naïve participants, showed a bias against choreographies that they believed were computer-generated, compared to choreographies they believed were human-generated (when in reality all choreographies presented were human-generated).

### 2.15 Qualitative data – experiments 1 and 2

We collected qualitative data in both experiments 1 and 2, which involved asking participants to answer questions in as much detail as they chose (see Methods section). We do not analyse these data formally as they were collected only for exploratory purposes, and to see whether participants' qualitative responses match to their quantitative data. Exploration of the qualitative data suggested that some participants had favourable attitudes toward computer-generated choreography, if computers were used as a supplement to choreography, as opposed to playing an independent role in dance choreography. Across both experiments, almost half of our participants (54.44%) reported that they valued computer-generated choreography, and that computer-generated dance could potentially serve a useful role in dance training, and enhancing human choreographic productions (e.g., “Yes, I do think it is valuable. It can be used to enhance and support the creative process. It could be used when someone has a dancers-block or to stimulate ideas for choreographies. It may also lead to new forms of visual art we haven't thought of yet”). However, 44.11% of participants thought that using computer-generated choreography in dance productions would mean an end to human creativity and did not believe in integrating computer-generated choreographies with human-generated ones (e.g., “… [I] feel it's a great threat to arts as it would mean people will be highly dependent only on this and would lose the ability to think and create more. My short answer, no I am not in support [of using computer-generated choreography].”). Overall, participants associated computer-generated choreographies with less fluency, smoothness, naturalness, familiarity, and continuity, both in Experiments 1 and 2. They also thought computer generated choreographies were more random, less sophisticated, less graceful, repetitive, and simplistic. Interestingly, even when participants saw human-generated choreographies but believed they were computer-generated (such as in Experiment 2), they assumed the choreographies were less natural or unfamiliar when they believed the choreographies were computer-generated (e.g., “… Maybe it was my bias that made me assume that computer generated choreographies were going to be a little brash, out of the box, aesthetically lacking.“). In keeping with our results from the quantitative data, participants reported that they liked a choreography more or found it more beautiful if they thought it flowed better and looked more natural (qualities they associated with human-generated choreographies in general).

### 2.16 Dance expertise – experiments 1 and 2

Scores on the dance expertise questionnaire (see Supplementary Material for the questions asked) were computed by summing individual scores on each item of the questionnaire. For Experiment 1, dance experts had on average 13.51 years of training in Bharatanatyam (SD = 8.41 years), and in Experiment 2, dance experts had on average 13.89 years of training (SD = 4.42 years). For both Experiments 1 and 2, one-tailed independent samples t-tests confirmed that dance experts scored higher on the dance expertise questionnaire (Experiment 1: Mean = 12.77, SD = 1.97; Experiment 2: Mean = 12.30, SD = 4.11) compared to non-experts (Experiment 1: Mean = 4.64, SD = 4.68; t (97) = 8.35, p < .001, 95% CI [6.52, ∞]; Experiment 2: Mean = 6.45, SD = 4.52; t (79) = 6.10, p < .001, 95% CI [4.26, ∞]).

## 3 General discussion

In the current study, across two pre-registered and statistically powered experiments, we aimed to shed light on the nature of aesthetic responses toward a particular type of computer-generated art by investigating observer prejudices against computer-generated dance choreography, and the impact of expertise and pre-conceived beliefs about the origin of artistic creation. Our results provide substantive evidence that an explicit bias exists among dance experts against computer-generated choreography, and the mere belief about a dance work's origin biases aesthetic responses toward artworks among both dance experts and dance naïve participants.

Specifically, as hypothesized, participants showed a bias against computer-generated dance choreography. However, the bias was present only for dance experts, and was explicit. That is, the bias existed only when dance experts were aware that some choreographies are computer-generated. This explicit bias persisted (and was heightened) in dance experts when they themselves categorised the choreographies as computer-vs. human-generated. Further, a pre-conceived belief about the origin of artistic creation also influenced aesthetic ratings. Participants, both dance experts and dance naïve participants, showed a bias against choreographies that they believed were computer-generated, compared to choreographies they believed were human-generated (when in reality all choreographies presented were human-generated).

Our results are in line with previous evidence that suggests a bias against computer-generated art across a range of art forms including music, poetry, and fine arts [6–11]. We expected a heightened explicit bias based on the assumption that participants who categorised the dance choreographies first would remember which choreographies they rated later, and would show a stronger bias against computer-generated dance choreography. In contrast, the participants who rated the dance choreographies first would assume that all choreographies were generated by a human. However, contrary to Chamberlain and colleagues [9], who found both an implicit and explicit bias against computer-generated paintings for art expert and art naïve participants, we found only an explicit bias amongst dance experts.

One explanation for this discrepancy could be that our participants, especially the non-experts, could not differentiate between computer- and human-generated choreographies. While accuracy was above chance level for both experts and non-experts for human-generated dance choreographies, it was below chance for computer-generated dance choreographies. These accuracy findings can in part be explained by the bias of participants to classify most choreographies as human generated. Lower accuracy also suggests that computer generated dance choreography (as created in the current study) cannot be easily detected from its visual and content characteristics alone. We found the same pattern of results, however, when participants themselves categorised the choreographies as human- or computer-generated. A bias was present only among dance experts who performed the categorisation task first. However, a visual inspection of the data suggests that when participants themselves categorised choreographies, the difference in aesthetic ratings of beauty, liking, and enjoyability for computer- and human-generated choreographies was more pronounced among experts and non-experts, although not significant as per our statistical threshold (see Fig. 4). An alternative explanation may be that dance as a performing art is processed differently to paintings and poetry. Indeed, dance involves not just a choreographer, but also a performer. In the case of computer-generated dance, the agent performing the dance choreography, at least in the context of the current experiments, is still a human agent. This might diminish the “machine” or “artificial” component for spectators, blurring the boundary between computer- and human-generated choreographies. An interesting avenue for future research would be to dissociate between artificial performers and artificial choreographers, and investigate to what extent these might affect observers’ aesthetic responses.

Supporting some previous findings suggesting modulation of the bias against computer-generated art [17], but in contrast to Chamberlain and colleagues [9]; only our dance experts showed a bias against computer-generated dance choreography in Experiment 1. Dance experts recruited in the current study had many years of training (an average of ∼13 years of training) and could therefore have a higher overall level of expertise than experts recruited in previous studies [9]. Our study does not test for a direction of the bias, i.e., it is unclear whether participants first categorise the dance choreography as computer- or human-generated, and then rate it as less or more aesthetically pleasing, or whether they first rate the choreography as more or less beautiful, and therefore think that it is computer- or human-generated based on their ratings. Models of aesthetic appreciation for fine arts distinguish between an initial, fast stage of processing, followed by a slower, more controlled process (Leder et al., 2004). Categorisation has also been found to be a fast, automatic process (e.g., Greene & Fei-Fei, 2014). However, an initial positive response in the current study for a particular dance choreography could have led to a faster aesthetic response followed by the categorisation, especially for participants who did the rating task first. This could explain a lack of differences in ratings between computer- and human-generated choreographies in both experts and non-experts. But when participants were explicitly aware that some choreographies were human- or computer-generated i.e., participants, especially dance experts, who did the categorisation task first, they possibly engaged in faster categorisation followed by the aesthetic responses. Indeed, [15] suggest that art experts might engage in different classification processes compared to art naïve participants, and perform more art-specific classifications.

In Experiment 2, both dance experts and dance naïve participants showed a bias against choreographies they believed were computer-generated (even when they were all, in reality, human-generated). This finding suggests that the belief about the origin of artistic creation modulates aesthetic ratings, and that top-down knowledge cues to the human origins of dance choreography influence both dance experts and dance naïve participants, overriding bottom-up stimulus perception. A growing body of research in social cognition supports the current findings – beliefs about the human origins of a stimulus can markedly modulate its perception ([[18], [19], [20]]). Of particular relevance, prior work has shown that actions believed to originate from human movements are rated as smoother and more enjoyable to watch than those originating from computer animation. The ratings of smoothness were unaffected by the agent form (i.e., whether the agent was a human or a robot; [20]. Our findings go a step further by demonstrating that even at the subtle level of dance choreography (and not just movement perception), aesthetic ratings of the choreography are modulated by the belief about the human origins of the creative production.

Indeed, an alternative explanation of our current findings relates to the exposure effects on aesthetic responses found in previous work. For instance, it is possible that computer-generated choreographies are rated as less beautiful because participants recognise computer-generated choreographies as being more random (as also suggested by our qualitative data). Previous research suggests that dance spectators can implicitly learn rules governing a dance choreography, and that these rules are important when making aesthetic judgements [[33], [37]]. Thus, our experts who are more familiar with the rules governing a dance style such as Bharatanatyam might be better able to identify random sequences vs. intentionally choreographed sequences, and their preferences might actually reflect a bias against random sequences (as opposed to the choreographic sequences originating from a computer). Thus, a mere exposure effect in terms of higher familiarity with the human-generated choreographies might explain a bias against the less familiar computer-generated choreographies. However, this explanation seems unlikely as we controlled for familiarity in the current analyses, and the source of choreography (i.e., whether computer- or human-generated) continued to predict aesthetic ratings above and beyond ratings of familiarity and reproducibility of the dance sequences.

It is important to note that across different art forms and creative productions, other characteristics of computer-generated art can be different to those of human-generated art. Future investigations will need to de-alienate whether a bias exists because computer-generated art is “random” and “less familiar” or whether a bias exists because the origin of the art is “computer-generated” or, as our results suggests, possibly a combination of both. In addition, the current experiments used choreographies that were randomly generated by a computer as opposed to choreographies created by artificial intelligence relying on specific rules of the dance style itself. An advantage of using Bharatanatyam was that none of the randomly generated sequences were wrong *per se* – i.e., they still made sense according to the vocabulary of the dance style, but these sequences would definitely be unlikely to appear in an actual dance performance. However, it is possible that some of these randomly generated choreographies, or choreographies generated “by chance”, more strongly resembled human-generated choreography than others, and might thus explain why we did not find an implicit difference between human- and computer-generated choreographies. As discussed in the introduction, chance has been used as a creative, structural, and philosophical principle in art for centuries, and has also been described as an element of unpredictability ([38]). Thus, our results may reflect differences between human-generated and chance-generated choreographies, instead of AI/computer-generated choreographies. However, results from Experiment 2, where participants believe that a choreography is human- or computer-generated (here, created by AI), still provide evidence for differences in how choreographies believed to be human- or computer-generated are perceived.

Future work using a dancer's choreographic style combined with AI algorithms should enable us to further investigate the role of source of choreography and its link to aesthetic responses. For instance, an artificial neural network can learn the structure and rules of Bharatanatyam Indian classical dance and generate choreographies [4]. Such choreographies can further motivate questions such as whether “learned” artificial choreographies are judged any differently from randomly-generated choreographies, and whether one is more (or less) human-like than the other.

Our work further highlights the importance of dance and dance research to understand human social and cognitive functions. Along with investigating dance for the sake of understanding dance from a psychological perspective [39], dance and dance expertise have already been valuable tools in trying to delineate functions of the sensorimotor system (e.g., [[20], [40]]). Dance, as explored in the current study, can also be a potential tool to understand social interactions (both human—human, and human—machine interactions), movement perception, reward and value judgements, and experience-dependent plasticity.

The theoretical implications of the current findings extend beyond the field of empirical aesthetics, and serve to inform several disciplines including artificial intelligence, engineering, robotics, and social cognition and neuroscience. Scientists and artists are pushing the boundaries of artistic AI, and designers are working on creating interactive artificial agents as similar to humans as possible (following the “like-me” hypothesis; [41]). The current findings suggest that merely how an artificial agent looks or is perceived goes beyond just the stimulus cues to its humanness. Perception (and aesthetic evaluation) of agents and their creative productions is largely dependent not only on knowledge about an agent's or production's human (or artificial) origins, but also the beliefs held about these origins. Such pre-conceived beliefs have large scale implications for artificial agents and their creative productions in the arts, as well as at schools, workplaces, care homes, and other social environments. Along with physical form and content of artificial agents and art productions, the viewers' knowledge and attitudes toward artistic AI and artificial agents will need to be taken into consideration for effective human-computer/human-AI interactions.

## 4 Conclusion

Results from the current study provide substantive evidence that an explicit bias exists among dance experts against computer-generated choreography, and the mere belief about a dance work's origin biases aesthetic responses toward artworks among both dance experts and dance naïve participants. In an article published on Venture Beat titled ‘This is how we'll merge with AI’, author and software developer Gary Grossman wrote “the relationship between humans and AI is something of a dance. We and AI come close together operating collaboratively, then are pushed away by the impossibility, only to stumble but return attracted by the potential.” [42]. It is only fitting that the dance community has started to embrace computer-generated choreography, and robots are sharing stage with human dancers. The findings from the current study have key implications not just for the future of artistic AI, and a better understanding of what constitutes the “human” and the “artificial,” but also for optimising artificial agents for effective human-computer/human-AI interactions in a world that is leading to a merging between humans and machines.
