# Supplementary material for: The computer, A choreographer? Aesthetic responses to randomly-generated dance choreography by a computer
Source: Heliyon. 2022 Dec 30;9(1):e12750. doi: 10.1016/j.heliyon.2022.e12750 (PMC9852657; doi:10.1016/j.heliyon.2022.e12750)
Supplement: Multimedia component 1 [file mmc1.docx]

Journal: …

**Supplementary Material**

The Computer, A Choreographer?

Aesthetic Responses to Computer-Generated Dance Choreography.

Kohinoor M. Darda^1,2,3^, Emily S. Cross^1,2^

^1^Institute of Neuroscience and Psychology, University of Glasgow, UK

^2^Department of Cognitive Science, Macquarie University, Sydney, Australia

^3^Penn Center for Neuroaesthetics, University of Pennsylvania, Philadelphia (PA), USA

Corresponding authors:

E-mail: [kohinoordarda@gmail.com](mailto:kohinoordarda@gmail.com), [emily.cross@glasgow.ac.uk](mailto:emily.cross@glasgow.ac.uk)

Table S1. Categorisation task accuracy – means and standard deviations across source of choreography, first task, and expertise.

| choreo | artexp | firsttask | mean_acc | sd_acc |
| --- | --- | --- | --- | --- |
| human generated | expert | ratefirst | 0.8533333 | 0.2385139 |
| human generated | expert | catfirst | 0.7666667 | 0.2652134 |
| human generated | nonexpert | ratefirst | 0.5802469 | 0.2652622 |
| human generated | nonexpert | catfirst | 0.5357143 | 0.2651079 |
| computer generated | expert | ratefirst | 0.5266667 | 0.2576547 |
| computer generated | expert | catfirst | 0.5000000 | 0.2902606 |
| computer generated | nonexpert | ratefirst | 0.4419753 | 0.2076718 |
| computer generated | nonexpert | catfirst | 0.3619048 | 0.2276584 |

Table S2. Categorisation task– 2 (source of choreography: human-generated, computer-generated) x 2 (expertise: experts, non-experts) x 2 (firsttask: ratefirst, catfirst) ANOVA results for accuracy as the dependent variable.

| Predictor | Sum  of  Squares | *df* | Mean  Square | *F* | *p* | _partial_ η^2^ | _partial_ η^2^  90% CI  [LL, UL] |
| --- | --- | --- | --- | --- | --- | --- | --- |
| (Intercept) | 14.56 | 1 | 14.56 | 227.83 | .000 |  |  |
| artexp | 0.86 | 1 | 0.86 | 13.40 | .000 | .07 | [.02, .13] |
| choreo | 1.07 | 1 | 1.07 | 16.69 | .000 | .08 | [.03, .15] |
| firsttask | 0.08 | 1 | 0.08 | 1.28 | .259 | .01 | [.00, .04] |
| artexp x choreo | 0.20 | 1 | 0.20 | 3.19 | .076 | .02 | [.00, .06] |
| artexp x firsttask | 0.01 | 1 | 0.01 | 0.17 | .682 | .00 | [.00, .02] |
| choreo x firsttask | 0.02 | 1 | 0.02 | 0.31 | .580 | .00 | [.00, .02] |
| artexp x choreo x firsttask | 0.03 | 1 | 0.03 | 0.43 | .511 | .00 | [.00, .03] |
| Error | 12.15 | 190 | 0.06 |  |  |  |  |

Table S3. Means and SDs for beauty, liking, enjoyability, familiarity, and reproducibility across source of choreography (human-generated, computer-generated), first task (RateFirst, CatFirst), expertise (experts, non-experts).

| FirstTask | Expertise | Choreo | Familiarity | Reproducibility | Beauty | Liking | Enjoyability | sd_fam | sd_reprod | sd_beauty | sd_liking | sd_enjoy |
| --- | --- | --- | --- | --- | --- | --- | --- | --- | --- | --- | --- | --- |
| ratefirst | expert | human generated | 4.20 | 1.61 | 3.58 | 3.46 | 3.49 | 1.08 | 0.98 | 1.06 | 1.05 | 1.06 |
| ratefirst | expert | computer generated | 3.04 | 2.06 | 3.29 | 3.28 | 3.26 | 1.39 | 1.10 | 1.02 | 1.07 | 1.02 |
| ratefirst | nonexpert | human generated | 2.21 | 2.84 | 2.87 | 2.82 | 2.84 | 1.32 | 1.23 | 1.15 | 1.18 | 1.16 |
| ratefirst | nonexpert | computer generated | 2.16 | 3.15 | 2.95 | 2.80 | 2.81 | 1.25 | 1.11 | 1.13 | 1.10 | 1.11 |
| catfirst | expert | human generated | 4.19 | 1.45 | 3.74 | 3.71 | 3.67 | 1.01 | 0.87 | 0.94 | 0.97 | 0.95 |
| catfirst | expert | computer generated | 2.78 | 1.93 | 2.84 | 2.79 | 2.83 | 1.43 | 1.07 | 1.12 | 1.12 | 1.16 |
| catfirst | nonexpert | human generated | 2.17 | 2.99 | 2.97 | 2.93 | 3.01 | 1.15 | 1.11 | 1.09 | 1.04 | 1.06 |
| catfirst | nonexpert | computer generated | 2.04 | 3.22 | 3.11 | 3.03 | 3.04 | 1.06 | 1.01 | 1.04 | 0.99 | 1.02 |

Table S4. Main models for beauty, liking, and enjoyability.

*Main model = clmm(beauty/liking/enjoyability ~ 1 + source of choreography*dance expertise + (1 + source of choreography* | *subject) + (1 + dance expertise* | *item), link = “logit”, threshold = “flexible”)*

|  | Main Model - Beauty | | | Main Model - Liking | | | Main Model - Enjoyability | | |
| --- | --- | --- | --- | --- | --- | --- | --- | --- | --- |
| **Predictors** | *Log-Odds* | *CI* | *p* | *Log-Odds* | *CI* | *p* | *Log-Odds* | *CI* | *p* |
| **1\|2** | -4.04 | -4.52 – -3.57 | **<0.001** | -3.75 | -4.20 – -3.30 | **<0.001** | -3.66 | -4.12 – -3.21 | **<0.001** |
| **2\|3** | -1.50 | -1.94 – -1.06 | **<0.001** | -1.31 | -1.73 – -0.89 | **<0.001** | -1.45 | -1.87 – -1.02 | **<0.001** |
| **3\|4** | 0.72 | 0.28 – 1.15 | **0.001** | 0.87 | 0.46 – 1.29 | **<0.001** | 0.82 | 0.39 – 1.24 | **<0.001** |
| **4\|5** | 3.21 | 2.75 – 3.66 | **<0.001** | 3.27 | 2.83 – 3.70 | **<0.001** | 3.27 | 2.83 – 3.71 | **<0.001** |
| **Dance Expertise** | 1.00 | 0.23 – 1.78 | **0.011** | 1.04 | 0.30 – 1.78 | **0.006** | 0.97 | 0.23 – 1.70 | **0.010** |
| **Source of Choreography** | 0.64 | 0.12 – 1.16 | **0.015** | 0.67 | 0.16 – 1.18 | **0.010** | 0.71 | 0.18 – 1.24 | **0.009** |
| **Dance Expertise by Source of Choreography** | 1.85 | 1.17 – 2.54 | **<0.001** | 1.50 | 0.77 – 2.23 | **<0.001** | 1.38 | 0.68 – 2.09 | **<0.001** |
| **Random Effects** | | | | | | | | | |
| σ^2^ | 3.29 | | | 3.29 | | | 3.29 | | |
| τ_00_ | 3.65 _sid_ | | | 3.22 _sid_ | | | 3.20 _sid_ | | |
|  | 0.31 _itemno_ | | | 0.28 _itemno_ | | | 0.34 _itemno_ | | |
| τ_11_ | 2.16 _sid.choreo.c_ | | | 2.48 _sid.choreo.c_ | | | 2.24 _sid.choreo.c_ | | |
|  | 0.08 _itemno.artexp.c_ | | | 0.11 _itemno.artexp.c_ | | | 0.13 _itemno.artexp.c_ | | |
| ρ_01_ | -0.04 _sid_ | | | -0.08 _sid_ | | | -0.10 _sid_ | | |
|  | 0.82 _itemno_ | | | 0.69 _itemno_ | | | 0.72 _itemno_ | | |
| ICC | 0.58 | | | 0.56 | | | 0.56 | | |
| N | 99 _sid_ | | | 99 _sid_ | | | 99 _sid_ | | |
|  | 30 _itemno_ | | | 30 _itemno_ | | | 30 _itemno_ | | |
| Observations | 2970 | | | 2970 | | | 2970 | | |
| Marginal R^2^ / Conditional R^2^ | 0.064 / 0.605 | | | 0.062 / 0.584 | | | 0.057 / 0.581 | | |
| AIC | 7107.213 | | | 7245.219 | | | 7235.578 | | |

Table S5. Subjective models for beauty, liking, and enjoyability.

*Subj model = clmm(beauty/liking/enjoyability ~ 1 + source of choreography*dance expertise + recruitment_platform + familiarity + reproducibility + (1 + source of choreography* | *subject) + (1 + dance expertise* | *item), link = “logit”, threshold = “flexible”)*

|  | Subj Model - Beauty | | | Subj Model - Liking | | | Subj Model - Enjoyability | | |
| --- | --- | --- | --- | --- | --- | --- | --- | --- | --- |
| Predictors | *Log-Odds* | *CI* | *p* | *Log-Odds* | *CI* | *p* | *Log-Odds* | *CI* | *p* |
| 1\|2 | -0.87 | -1.42 – -0.31 | **0.002** | -0.62 | -1.16 – -0.08 | **0.025** | -0.49 | -1.04 – 0.06 | 0.078 |
| 2\|3 | 1.76 | 1.21 – 2.30 | **<0.001** | 1.90 | 1.37 – 2.43 | **<0.001** | 1.81 | 1.27 – 2.35 | **<0.001** |
| 3\|4 | 4.11 | 3.54 – 4.67 | **<0.001** | 4.21 | 3.65 – 4.76 | **<0.001** | 4.21 | 3.66 – 4.77 | **<0.001** |
| 4\|5 | 6.73 | 6.13 – 7.33 | **<0.001** | 6.73 | 6.14 – 7.31 | **<0.001** | 6.80 | 6.21 – 7.40 | **<0.001** |
| Dance Expertise | 0.21 | -0.67 – 1.08 | 0.642 | 0.48 | -0.36 – 1.33 | 0.261 | 0.46 | -0.39 – 1.30 | 0.292 |
| Source of Choreography | 0.49 | 0.03 – 0.95 | **0.039** | 0.49 | 0.03 – 0.96 | **0.037** | 0.53 | 0.05 – 1.02 | **0.032** |
| Familiarity | 0.58 | 0.48 – 0.68 | **<0.001** | 0.61 | 0.51 – 0.71 | **<0.001** | 0.62 | 0.52 – 0.71 | **<0.001** |
| Reproducibility | 0.60 | 0.50 – 0.70 | **<0.001** | 0.58 | 0.48 – 0.68 | **<0.001** | 0.60 | 0.51 – 0.70 | **<0.001** |
| Recruitment Platform | -1.30 | -2.20 – -0.40 | **0.005** | -0.83 | -1.69 – 0.04 | 0.062 | -0.77 | -1.64 – 0.11 | 0.085 |
| Dance Expertise by Source of Choreography | 1.36 | 0.68 – 2.03 | **<0.001** | 0.95 | 0.25 – 1.65 | **0.008** | 0.83 | 0.13 – 1.53 | **0.020** |
| **Random Effects** | | | | | | | | | |
| σ^2^ | 3.29 | | | 3.29 | | | 3.29 | | |
| τ_00_ | 2.58 _sid_ | | | 2.38 _sid_ | | | 2.38 _sid_ | | |
|  | 0.23 _itemno_ | | | 0.22 _itemno_ | | | 0.27 _itemno_ | | |
| τ_11_ | 1.77 _sid.choreo.c_ | | | 1.97 _sid.choreo.c_ | | | 1.87 _sid.choreo.c_ | | |
|  | 0.16 _itemno.artexp.c_ | | | 0.16 _itemno.artexp.c_ | | | 0.19 _itemno.artexp.c_ | | |
| ρ_01_ | 0.07 _sid_ | | | 0.04 _sid_ | | | 0.00 _sid_ | | |
|  | 1.00 _itemno_ | | | 1.00 _itemno_ | | | 0.94 _itemno_ | | |
| ICC | 0.50 | | | 0.49 | | | 0.49 | | |
| N | 99 _sid_ | | | 99 _sid_ | | | 99 _sid_ | | |
|  | 30 _itemno_ | | | 30 _itemno_ | | | 30 _itemno_ | | |
| Observations | 2970 | | | 2970 | | | 2970 | | |
| Marginal R^2^ / Conditional R^2^ | 0.238 / 0.618 | | | 0.218 / 0.598 | | | 0.214 / 0.598 | | |
| AIC | 6841.111 | | | 6980.322 | | | 6961.300 | | |

Table S6. Maximal models for beauty, liking, and enjoyability.

*Max model = clmm(beauty/liking/enjoyability ~ 1 + source of choreography*dance expertise*firsttask + (1 + source of choreography* | *subject) + (1 + dance expertise * firsttask* | *item), link = “logit”, threshold = “flexible”)*

|  | Max Model - Beauty | | | Max Model - Liking | | | Max Model - Enjoyability | | |
| --- | --- | --- | --- | --- | --- | --- | --- | --- | --- |
| Predictors | *Log-Odds* | *CI* | *p* | *Log-Odds* | *CI* | *p* | *Log-Odds* | *CI* | *p* |
| 1\|2 | -4.07 | -4.54 – -3.59 | **<0.001** | -3.77 | -4.22 – -3.32 | **<0.001** | -3.69 | -4.14 – -3.23 | **<0.001** |
| 2\|3 | -1.51 | -1.96 – -1.07 | **<0.001** | -1.32 | -1.74 – -0.90 | **<0.001** | -1.46 | -1.89 – -1.03 | **<0.001** |
| 3\|4 | 0.71 | 0.27 – 1.15 | **0.001** | 0.87 | 0.46 – 1.29 | **<0.001** | 0.82 | 0.39 – 1.24 | **<0.001** |
| 4\|5 | 3.20 | 2.75 – 3.66 | **<0.001** | 3.27 | 2.83 – 3.70 | **<0.001** | 3.27 | 2.83 – 3.72 | **<0.001** |
| Dance Expertise | 1.02 | 0.24 – 1.80 | **0.010** | 1.06 | 0.33 – 1.80 | **0.005** | 0.99 | 0.25 – 1.72 | **0.009** |
| Source of Choreography | 0.61 | 0.10 – 1.11 | **0.019** | 0.63 | 0.13 – 1.13 | **0.013** | 0.68 | 0.15 – 1.20 | **0.011** |
| FirstTask | -0.09 | -0.87 – 0.68 | 0.816 | -0.00 | -0.73 – 0.72 | 0.990 | 0.03 | -0.70 – 0.75 | 0.946 |
| Dance Expertise by Source of Choreography | 1.79 | 1.12 – 2.45 | **<0.001** | 1.42 | 0.73 – 2.10 | **<0.001** | 1.32 | 0.63 – 2.00 | **<0.001** |
| Dance Expertise by First Task | -0.68 | -2.22 – 0.86 | 0.388 | -0.71 | -2.16 – 0.74 | 0.336 | -0.75 | -2.20 – 0.70 | 0.310 |
| Source of Choreography by First Task | 0.70 | 0.06 – 1.33 | **0.033** | 0.77 | 0.11 – 1.43 | **0.022** | 0.64 | -0.01 – 1.29 | 0.053 |
| Dance Expertise by Source of Choreography by FirstTask | 1.66 | 0.40 – 2.91 | **0.010** | 2.18 | 0.87 – 3.48 | **0.001** | 1.72 | 0.43 – 3.01 | **0.009** |
| **Random Effects** | | | | | | | | | |
| σ^2^ | 3.29 | | | 3.29 | | | 3.29 | | |
| τ_00_ | 3.63 _sid_ | | | 3.19 _sid_ | | | 3.18 _sid_ | | |
|  | 0.31 _itemno_ | | | 0.28 _itemno_ | | | 0.34 _itemno_ | | |
| τ_11_ | 1.92 _sid.choreo.c_ | | | 2.10 _sid.choreo.c_ | | | 2.00 _sid.choreo.c_ | | |
|  | 0.09 _itemno.artexp.c_ | | | 0.11 _itemno.artexp.c_ | | | 0.14 _itemno.artexp.c_ | | |
|  | 0.04 _itemno.firsttask.c_ | | | 0.03 _itemno.firsttask.c_ | | | 0.04 _itemno.firsttask.c_ | | |
|  | 0.04 _itemno.artexp.c:firsttask.c_ | | | 0.06 _itemno.artexp.c:firsttask.c_ | | | 0.11 _itemno.artexp.c:firsttask.c_ | | |
| ρ_01_ | -0.01 _sid_ | | | -0.04 _sid_ | | | -0.07 _sid_ | | |
|  | 0.76 _itemno.artexp.c_ | | | 0.67 _itemno.artexp.c_ | | | 0.69 _itemno.artexp.c_ | | |
|  | 0.39 _itemno.firsttask.c_ | | | 0.13 _itemno.firsttask.c_ | | | 0.13 _itemno.firsttask.c_ | | |
|  | 0.23 _itemno.artexp.c:firsttask.c_ | | | -0.47 _itemno.artexp.c:firsttask.c_ | | | -0.08 _itemno.artexp.c:firsttask.c_ | | |
| ICC | 0.57 | | | 0.55 | | | 0.55 | | |
| N | 99 _sid_ | | | 99 _sid_ | | | 99 _sid_ | | |
|  | 30 _itemno_ | | | 30 _itemno_ | | | 30 _itemno_ | | |
| Observations | 2970 | | | 2970 | | | 2970 | | |
| Marginal R^2^ / Conditional R^2^ | 0.075 / 0.606 | | | 0.079 / 0.585 | | | 0.070 / 0.583 | | |
| AIC | 7115.416 | | | 7249.913 | | | 7240.443 | | |

Table S7. Maximal subjective models for beauty, liking, and enjoyability.

*Max subj model = clmm(beauty/liking ~ 1 + source of choreography*dance expertise*firsttask + familiarity + reproducibility + recruitment_platform + (1 + source of choreography* | *subject) + (1 + dance expertise * firsttask* | *item), link = “logit”, threshold = “flexible”)*

|  | Max Subj Model - Beauty | | | Max Subj Model - Liking | | | Max Subj Model - Enjoyability | | |
| --- | --- | --- | --- | --- | --- | --- | --- | --- | --- |
| Predictors | *Log-Odds* | *CI* | *p* | *Log-Odds* | *CI* | *p* | *Log-Odds* | *CI* | *p* |
| 1\|2 | -0.86 | -1.42 – -0.30 | **0.002** | -0.59 | -1.14 – -0.05 | **0.032** | -0.50 | -1.05 – 0.05 | 0.075 |
| 2\|3 | 1.78 | 1.23 – 2.32 | **<0.001** | 1.94 | 1.40 – 2.47 | **<0.001** | 1.82 | 1.28 – 2.36 | **<0.001** |
| 3\|4 | 4.13 | 3.57 – 4.69 | **<0.001** | 4.25 | 3.70 – 4.81 | **<0.001** | 4.24 | 3.68 – 4.80 | **<0.001** |
| 4\|5 | 6.76 | 6.16 – 7.36 | **<0.001** | 6.78 | 6.19 – 7.37 | **<0.001** | 6.84 | 6.24 – 7.43 | **<0.001** |
| Dance Expertise | 0.22 | -0.65 – 1.09 | 0.621 | 0.49 | -0.35 – 1.33 | 0.249 | 0.46 | -0.38 – 1.31 | 0.285 |
| Source of Choreography | 0.46 | 0.01 – 0.91 | **0.047** | 0.45 | 0.01 – 0.90 | **0.046** | 0.50 | 0.02 – 0.98 | **0.039** |
| FirstTask | -0.00 | -0.66 – 0.66 | 0.996 | 0.09 | -0.54 – 0.72 | 0.783 | 0.12 | -0.51 – 0.76 | 0.705 |
| Familiarity | 0.58 | 0.48 – 0.68 | **<0.001** | 0.62 | 0.52 – 0.72 | **<0.001** | 0.62 | 0.52 – 0.72 | **<0.001** |
| Reproducibility | 0.61 | 0.51 – 0.71 | **<0.001** | 0.59 | 0.49 – 0.68 | **<0.001** | 0.61 | 0.51 – 0.70 | **<0.001** |
| Recruitment Platform | -1.31 | -2.21 – -0.42 | **0.004** | -0.82 | -1.69 – 0.04 | 0.061 | -0.79 | -1.65 – 0.08 | 0.076 |
| Dance Expertise by Source of Choreography | 1.29 | 0.64 – 1.93 | **<0.001** | 0.86 | 0.20 – 1.52 | **0.011** | 0.76 | 0.09 – 1.44 | **0.027** |
| Dance Expertise by First Task | -0.53 | -1.84 – 0.77 | 0.424 | -0.57 | -1.83 – 0.70 | 0.379 | -0.63 | -1.89 – 0.63 | 0.328 |
| Source of Choreography by First Task | 0.63 | 0.03 – 1.22 | **0.039** | 0.69 | 0.09 – 1.29 | **0.025** | 0.58 | -0.03 – 1.19 | 0.062 |
| Dance Expertise by Source of Choreography by FirstTask | 1.74 | 0.60 – 2.88 | **0.003** | 2.23 | 1.05 – 3.41 | **<0.001** | 1.81 | 0.61 – 3.01 | **0.003** |
| **Random Effects** | | | | | | | | | |
| σ^2^ | 3.29 | | | 3.29 | | | 3.29 | | |
| τ_00_ | 2.56 _sid_ | | | 2.36 _sid_ | | | 2.36 _sid_ | | |
|  | 0.23 _itemno_ | | | 0.21 _itemno_ | | | 0.27 _itemno_ | | |
| τ_11_ | 1.52 _sid.choreo.c_ | | | 1.60 _sid.choreo.c_ | | | 1.64 _sid.choreo.c_ | | |
|  | 0.15 _itemno.artexp.c_ | | | 0.17 _itemno.artexp.c_ | | | 0.21 _itemno.artexp.c_ | | |
|  | 0.05 _itemno.firsttask.c_ | | | 0.05 _itemno.firsttask.c_ | | | 0.06 _itemno.firsttask.c_ | | |
|  | 0.00 _itemno.artexp.c:firsttask.c_ | | | 0.08 _itemno.artexp.c:firsttask.c_ | | | 0.14 _itemno.artexp.c:firsttask.c_ | | |
| ρ_01_ | 0.11 _sid_ | | | 0.09 _sid_ | | | 0.03 _sid_ | | |
|  | 1.00 _itemno.artexp.c_ | | | 0.98 _itemno.artexp.c_ | | | 0.91 _itemno.artexp.c_ | | |
|  | 0.57 _itemno.firsttask.c_ | | | 0.40 _itemno.firsttask.c_ | | | 0.31 _itemno.firsttask.c_ | | |
|  | 0.34 _itemno.artexp.c:firsttask.c_ | | | -0.74 _itemno.artexp.c:firsttask.c_ | | | -0.22 _itemno.artexp.c:firsttask.c_ | | |
| ICC | 0.49 | | | 0.48 | | | 0.48 | | |
| N | 99 _sid_ | | | 99 _sid_ | | | 99 _sid_ | | |
|  | 30 _itemno_ | | | 30 _itemno_ | | | 30 _itemno_ | | |
| Observations | 2970 | | | 2970 | | | 2970 | | |
| Marginal R^2^ / Conditional R^2^ | 0.251 / 0.621 | | | 0.236 / 0.602 | | | 0.228 / 0.602 | | |
| AIC | 6846.845 | | | 6979.653 | | | 6964.412 | | |

Table S8. Maximal subjective models for beauty, liking, and enjoyability when source of choreography is categorised by participants.

*Max subj model = clmm(beauty/liking ~ 1 + source of choreography*dance expertise*firsttask + familiarity + reproducibility + recruitment_platform + (1 + source of choreography* | *subject) + (1 + dance expertise * firsttask* | *item), link = “logit”, threshold = “flexible”)*

|  | Max Subj Model - Beauty (Ppt) | | | Max Subj Model - Liking (Ppt) | | | Max Subj Model - Enjoyability (Ppt) | | |
| --- | --- | --- | --- | --- | --- | --- | --- | --- | --- |
| Predictors | *Log-Odds* | *CI* | *p* | *Log-Odds* | *CI* | *p* | *Log-Odds* | *CI* | *p* |
| 1\|2 | -0.73 | -1.27 – -0.18 | **0.009** | -0.40 | -0.91 – 0.12 | 0.136 | -0.41 | -0.94 – 0.12 | 0.130 |
| 2\|3 | 1.91 | 1.38 – 2.44 | **<0.001** | 2.10 | 1.59 – 2.61 | **<0.001** | 1.87 | 1.35 – 2.39 | **<0.001** |
| 3\|4 | 4.22 | 3.67 – 4.77 | **<0.001** | 4.35 | 3.82 – 4.89 | **<0.001** | 4.23 | 3.69 – 4.77 | **<0.001** |
| 4\|5 | 6.77 | 6.19 – 7.35 | **<0.001** | 6.80 | 6.23 – 7.36 | **<0.001** | 6.74 | 6.16 – 7.31 | **<0.001** |
| Dance Expertise | 0.15 | -0.71 – 1.01 | 0.734 | 0.40 | -0.42 – 1.22 | 0.339 | 0.42 | -0.40 – 1.24 | 0.315 |
| Source of Choreography (Participant) | 0.62 | 0.36 – 0.89 | **<0.001** | 0.46 | 0.18 – 0.75 | **0.001** | 0.42 | 0.16 – 0.69 | **0.002** |
| FirstTask | -0.09 | -0.74 – 0.56 | 0.795 | 0.00 | -0.61 – 0.62 | 0.988 | 0.04 | -0.58 – 0.65 | 0.907 |
| Familiarity | 0.62 | 0.52 – 0.71 | **<0.001** | 0.67 | 0.58 – 0.77 | **<0.001** | 0.65 | 0.55 – 0.74 | **<0.001** |
| Reproducibility | 0.60 | 0.50 – 0.69 | **<0.001** | 0.57 | 0.48 – 0.66 | **<0.001** | 0.57 | 0.48 – 0.67 | **<0.001** |
| Recruitment Platform | -1.16 | -2.04 – -0.28 | **0.009** | -0.68 | -1.52 – 0.15 | 0.110 | -0.64 | -1.48 – 0.19 | 0.130 |
| Dance Expertise by Source of Choreography (Participant) | 0.35 | -0.18 – 0.88 | 0.200 | 0.30 | -0.27 – 0.86 | 0.300 | 0.15 | -0.38 – 0.69 | 0.568 |
| Dance Expertise by First Task | -0.66 | -1.96 – 0.65 | 0.326 | -0.75 | -1.97 – 0.47 | 0.226 | -0.78 | -2.01 – 0.46 | 0.218 |
| Source of Choreography (Participant) by First Task | 0.45 | -0.08 – 0.98 | 0.095 | 0.59 | 0.03 – 1.14 | **0.037** | 0.57 | 0.04 – 1.09 | **0.035** |
| Dance Expertise by Source of Choreography (Participant) by FirstTask | 0.95 | -0.09 – 2.00 | 0.074 | 1.11 | 0.01 – 2.21 | **0.047** | 1.00 | -0.05 – 2.05 | 0.062 |
| **Random Effects** | | | | | | | | | |
| σ^2^ | 3.29 | | | 3.29 | | | 3.29 | | |
| τ_00_ | 2.46 _sid_ | | | 2.19 _sid_ | | | 2.18 _sid_ | | |
|  | 0.19 _itemno_ | | | 0.00 _itemno_ | | | 0.24 _itemno_ | | |
|  |  | | | 0.18 _itemno_ | | |  | | |
| τ_11_ | 1.01 _sid.choreo_ppt.c_ | | | 1.25 _sid.choreo_ppt.c_ | | | 1.01 _sid.choreo_ppt.c_ | | |
|  | 0.25 _itemno.artexp.c_ | | | 0.03 _itemno.cond.c_ | | | 0.22 _itemno.artexp.c_ | | |
|  | 0.06 _itemno.cond.c_ | | | 0.19 _itemno.artexp.c_ | | | 0.04 _itemno.cond.c_ | | |
|  | 0.40 _itemno.artexp.c:cond.c_ | | |  | | | 0.42 _itemno.artexp.c:cond.c_ | | |
| ρ_01_ | -0.10 _sid_ | | | 0.12 _sid_ | | | 0.08 _sid_ | | |
|  | 0.92 _itemno.artexp.c_ | | | -1.00 _itemno_ | | | 0.95 _itemno.artexp.c_ | | |
|  | 0.58 _itemno.cond.c_ | | | 1.00 _itemno_ | | | 0.17 _itemno.cond.c_ | | |
|  | 0.19 _itemno.artexp.c:cond.c_ | | |  | | | -0.07 _itemno.artexp.c:cond.c_ | | |
| ICC | 0.47 | | | 0.44 | | | 0.46 | | |
| N | 99 _sid_ | | | 99 _sid_ | | | 99 _sid_ | | |
|  | 30 _itemno_ | | | 30 _itemno_ | | | 30 _itemno_ | | |
| Observations | 2970 | | | 2970 | | | 2970 | | |
| Marginal R^2^ / Conditional R^2^ | 0.241 / 0.600 | | | 0.237 / 0.570 | | | 0.217 / 0.575 | | |
| AIC | 6898.955 | | | 7051.643 | | | 7042.534 | | |

Table S9. Experiment 2. Main models for beauty and liking when the participants believe that the choreography is human- or computer-generated – for all participants, as well as when excluding participants who did not fall for our belief manipulation (“belief”).

*Main model = clmm(beauty/liking ~ 1 + source of choreography*dance expertise + (1 + source of choreography* | *subject) + (1 + dance expertise* | *item), link = “logit”, threshold = “flexible”)*

|  | Main Model - Beauty | | | Main Model - Liking | | | Main Model - Beauty (Belief) | | | Main Model - Liking (Belief) | | |
| --- | --- | --- | --- | --- | --- | --- | --- | --- | --- | --- | --- | --- |
| **Predictors** | *Log-Odds* | *CI* | *p* | *Log-Odds* | *CI* | *p* | *Log-Odds* | *CI* | *p* | *Log-Odds* | *CI* | *p* |
| **1\|2** | -4.41 | -5.00 – -3.82 | **<0.001** | -4.72 | -5.35 – -4.10 | **<0.001** | -4.61 | -5.37 – -3.86 | **<0.001** | -4.92 | -5.72 – -4.13 | **<0.001** |
| **2\|3** | -2.22 | -2.72 – -1.72 | **<0.001** | -2.15 | -2.66 – -1.64 | **<0.001** | -2.21 | -2.82 – -1.60 | **<0.001** | -2.21 | -2.86 – -1.57 | **<0.001** |
| **3\|4** | -0.02 | -0.50 – 0.46 | 0.935 | -0.07 | -0.56 – 0.42 | 0.787 | -0.01 | -0.59 – 0.58 | 0.977 | 0.00 | -0.62 – 0.62 | 0.997 |
| **4\|5** | 2.24 | 1.74 – 2.73 | **<0.001** | 2.29 | 1.78 – 2.80 | **<0.001** | 2.24 | 1.62 – 2.85 | **<0.001** | 2.33 | 1.68 – 2.97 | **<0.001** |
| **Dance Expertise** | 0.34 | -0.35 – 1.02 | 0.337 | 0.39 | -0.29 – 1.06 | 0.265 | 0.61 | -0.35 – 1.57 | 0.211 | 0.55 | -0.42 – 1.53 | 0.265 |
| **Source of Choreography** | 0.56 | 0.29 – 0.84 | **<0.001** | 0.29 | 0.01 – 0.57 | **0.039** | 0.61 | 0.25 – 0.97 | **0.001** | 0.24 | -0.16 – 0.64 | 0.240 |
| **Dance Expertise by Source of Choreography** | -0.32 | -0.87 – 0.23 | 0.256 | -0.35 | -0.91 – 0.20 | 0.214 | -0.38 | -1.09 – 0.33 | 0.293 | -0.59 | -1.38 – 0.21 | 0.148 |
| **Random Effects** | | | | | | | | | | | | |
| σ^2^ | 3.29 | | | 3.29 | | | 3.29 | | | 3.29 | | |
| τ_00_ | 2.16 _sid_ | | | 1.98 _sid_ | | | 2.59 _sid_ | | | 2.68 _sid_ | | |
|  | 0.41 _itemno_ | | | 0.50 _itemno_ | | | 0.42 _itemno_ | | | 0.54 _itemno_ | | |
| τ_11_ | 0.56 _sid.choreo.c_ | | | 0.58 _sid.choreo.c_ | | | 0.59 _sid.choreo.c_ | | | 0.99 _sid.choreo.c_ | | |
|  | 0.04 _itemno.artexp.c_ | | | 0.14 _itemno.artexp.c_ | | | 0.07 _itemno.artexp.c_ | | | 0.07 _itemno.artexp.c_ | | |
| ρ_01_ | 0.03 _sid_ | | | 0.36 _sid_ | | | 0.17 _sid_ | | | 0.32 _sid_ | | |
|  | -0.51 _itemno_ | | | -0.56 _itemno_ | | | -0.53 _itemno_ | | | -1.00 _itemno_ | | |
| ICC | 0.45 | | | 0.45 | | | 0.49 | | | 0.51 | | |
| N | 81 _sid_ | | | 81 _sid_ | | | 49 _sid_ | | | 49 _sid_ | | |
|  | 15 _itemno_ | | | 15 _itemno_ | | | 15 _itemno_ | | | 15 _itemno_ | | |
| Observations | 1215 | | | 1215 | | | 735 | | | 735 | | |
| Marginal R^2^ / Conditional R^2^ | 0.018 / 0.463 | | | 0.011 / 0.452 | | | 0.029 / 0.505 | | | 0.016 / 0.521 | | |
| AIC | 3080.673 | | | 3064.183 | | | 1858.343 | | | 1838.878 | | |

Table S10. Experiment 2. Subjective models for beauty and liking when the participants believe that the choreography is human- or computer-generated – for all participants, as well as when excluding participants who did not fall for our belief manipulation (“belief”).

*Subj model = clmm(beauty/liking ~ 1 + source of choreography*dance expertise + recruitment platform + (1 + source of choreography* | *subject) + (1 + dance expertise* | *item), link = “logit”, threshold = “flexible”)*

|  | Subj Model - Beauty | | | Subj Model - Liking | | | Subj Model - Beauty (Belief) | | | Subj Model - Liking (Belief | | |
| --- | --- | --- | --- | --- | --- | --- | --- | --- | --- | --- | --- | --- |
| Predictors | *Log-Odds* | *CI* | *p* | *Log-Odds* | *CI* | *p* | *Log-Odds* | *CI* | *p* | *Log-Odds* | *CI* | *p* |
| **1\|2** | -4.38 | -4.99 – -3.77 | **<0.001** | -4.68 | -5.33 – -4.04 | **<0.001** | -4.67 | -5.48 – -3.85 | **<0.001** | -4.96 | -5.81 – -4.10 | **<0.001** |
| **2\|3** | -2.19 | -2.72 – -1.66 | **<0.001** | -2.11 | -2.65 – -1.57 | **<0.001** | -2.26 | -2.95 – -1.58 | **<0.001** | -2.24 | -2.96 – -1.53 | **<0.001** |
| **3\|4** | 0.01 | -0.50 – 0.52 | 0.974 | -0.03 | -0.55 – 0.49 | 0.919 | -0.06 | -0.72 – 0.60 | 0.856 | -0.03 | -0.72 – 0.66 | 0.930 |
| **4\|5** | 2.27 | 1.74 – 2.79 | **<0.001** | 2.33 | 1.79 – 2.87 | **<0.001** | 2.18 | 1.50 – 2.87 | **<0.001** | 2.30 | 1.58 – 3.01 | **<0.001** |
| **Dance Expertise** | 0.23 | -0.74 – 1.19 | 0.644 | 0.23 | -0.71 – 1.17 | 0.635 | 0.75 | -0.49 – 1.98 | 0.236 | 0.63 | -0.62 – 1.89 | 0.320 |
| **Source of Choreography** | 0.56 | 0.29 – 0.84 | **<0.001** | 0.29 | 0.02 – 0.57 | **0.038** | 0.61 | 0.25 – 0.97 | **0.001** | 0.24 | -0.16 – 0.64 | 0.241 |
| **Recruitment Platform** | -0.17 | -1.20 – 0.87 | 0.755 | -0.24 | -1.23 – 0.75 | 0.634 | 0.24 | -1.15 – 1.63 | 0.735 | 0.15 | -1.25 – 1.54 | 0.838 |
| **Dance Expertise by Source of Choreography** | -0.32 | -0.87 – 0.23 | 0.257 | -0.35 | -0.91 – 0.20 | 0.210 | -0.38 | -1.09 – 0.33 | 0.293 | -0.59 | -1.38 – 0.21 | 0.148 |
| **Random Effects** | | | | | | | | | | | | |
| σ^2^ | 3.29 | | | 3.29 | | | 3.29 | | | 3.29 | | |
| τ_00_ | 2.16 _sid_ | | | 1.97 _sid_ | | | 2.59 _sid_ | | | 2.68 _sid_ | | |
|  | 0.41 _itemno_ | | | 0.50 _itemno_ | | | 0.41 _itemno_ | | | 0.54 _itemno_ | | |
| τ_11_ | 0.56 _sid.choreo.c_ | | | 0.58 _sid.choreo.c_ | | | 0.60 _sid.choreo.c_ | | | 0.99 _sid.choreo.c_ | | |
|  | 0.04 _itemno.artexp.c_ | | | 0.14 _itemno.artexp.c_ | | | 0.07 _itemno.artexp.c_ | | | 0.07 _itemno.artexp.c_ | | |
| ρ_01_ | 0.03 _sid_ | | | 0.35 _sid_ | | | 0.17 _sid_ | | | 0.32 _sid_ | | |
|  | -0.51 _itemno_ | | | -0.56 _itemno_ | | | -0.53 _itemno_ | | | -1.00 _itemno_ | | |
| ICC | 0.45 | | | 0.45 | | | 0.49 | | | 0.51 | | |
| N | 81 _sid_ | | | 81 _sid_ | | | 49 _sid_ | | | 49 _sid_ | | |
|  | 15 _itemno_ | | | 15 _itemno_ | | | 15 _itemno_ | | | 15 _itemno_ | | |
| Observations | 1215 | | | 1215 | | | 735 | | | 735 | | |
| Marginal R^2^ / Conditional R^2^ | 0.019 / 0.463 | | | 0.012 / 0.452 | | | 0.029 / 0.505 | | | 0.016 / 0.521 | | |
| AIC | 3082.576 | | | 3065.956 | | | 1860.228 | | | 1840.836 | | |

Figure S1. Mean ratings of familiarity across source of choreography (human-generated, computer-generated), first task (RateFirst, CatFirst), expertise (experts, non-experts).


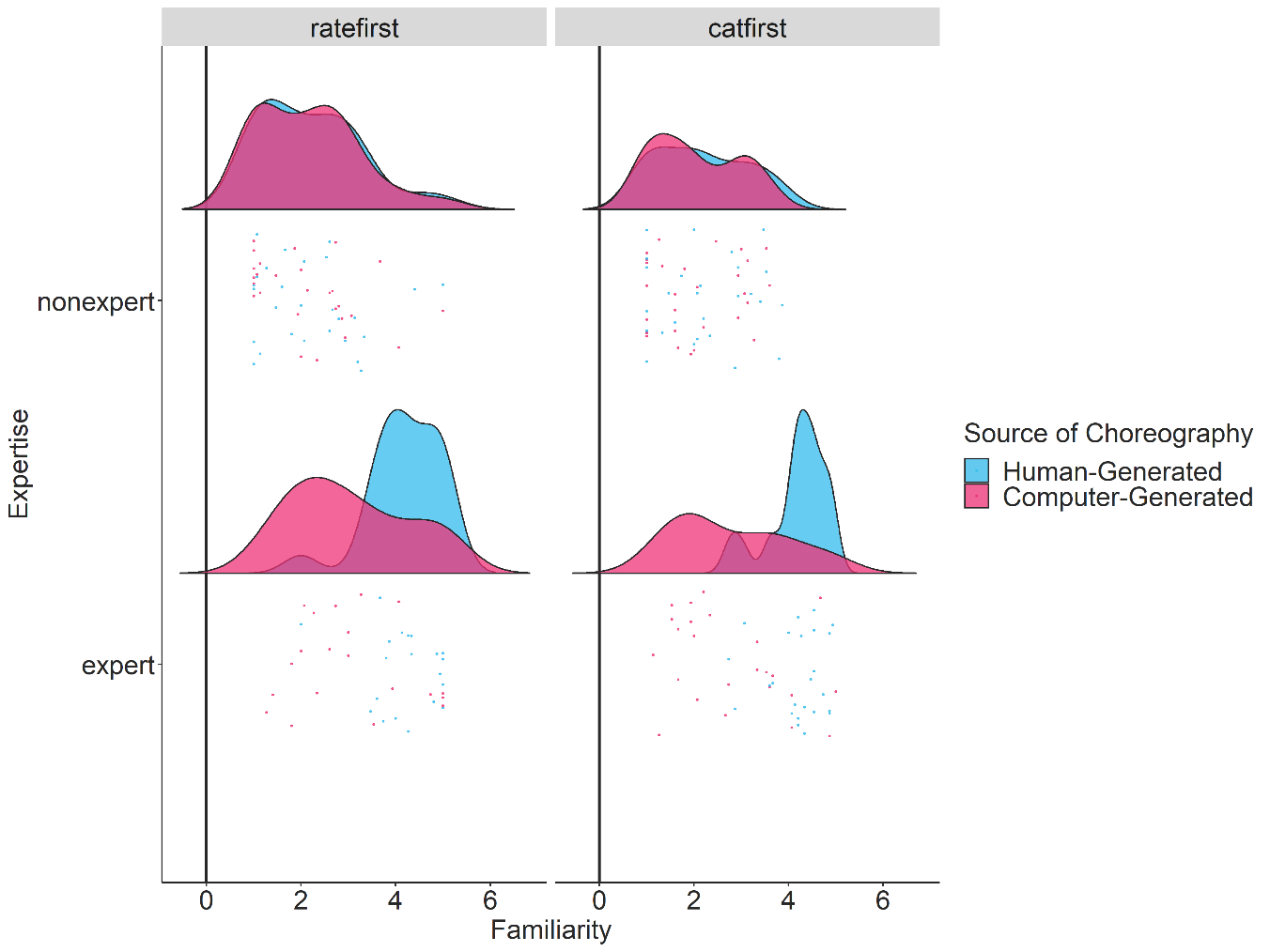


Figure S2. Mean ratings of reproducibility across source of choreography (human-generated, computer-generated), first task (RateFirst, CatFirst), expertise (experts, non-experts).


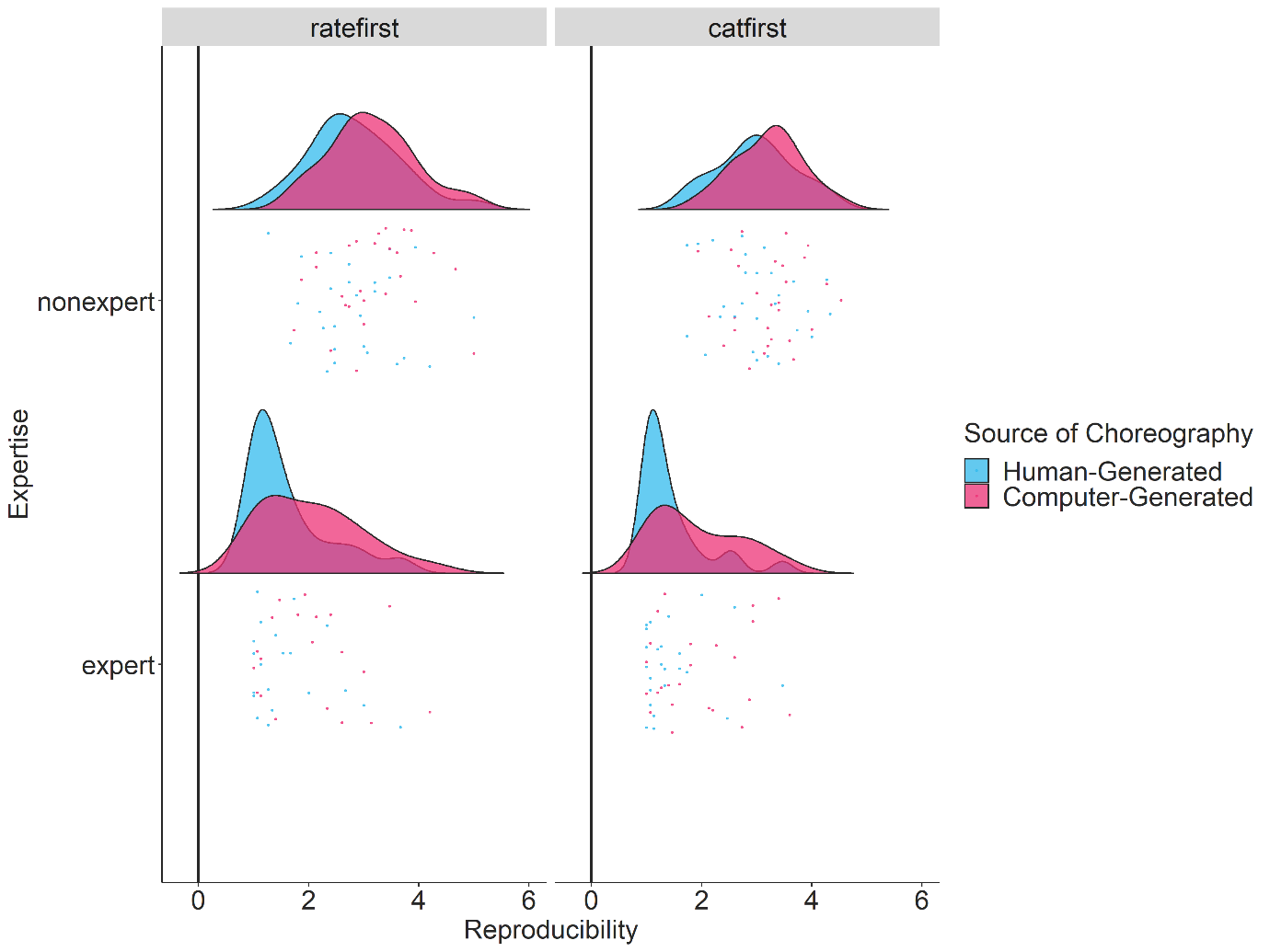


Figure S3. Mean ratings of enjoyability across source of choreography (human-generated, computer-generated), first task (RateFirst, CatFirst), expertise (experts, non-experts).


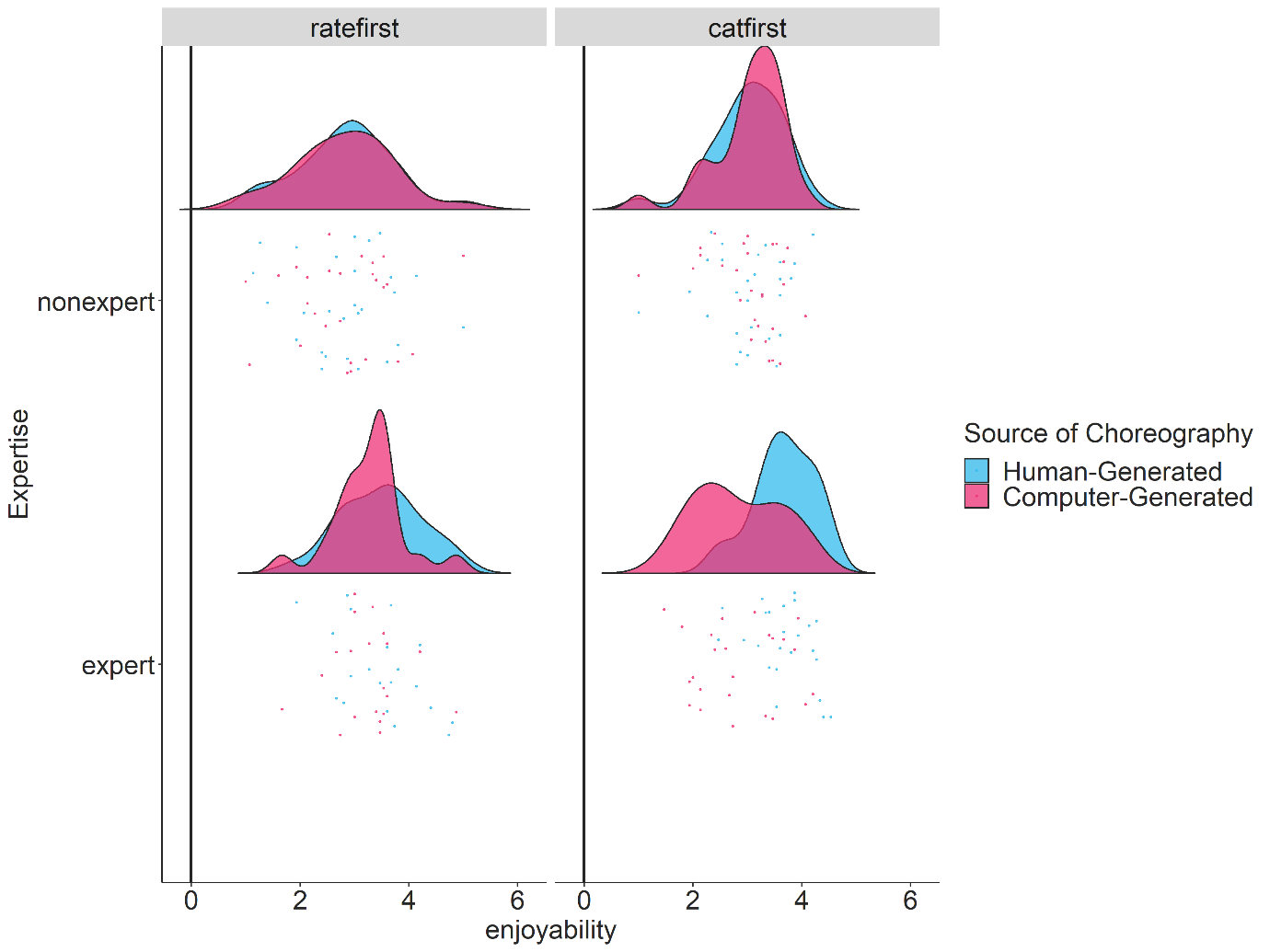


Figure S4. Mean ratings of liking across source of choreography (human-generated, computer-generated), first task (RateFirst, CatFirst), expertise (experts, non-experts).


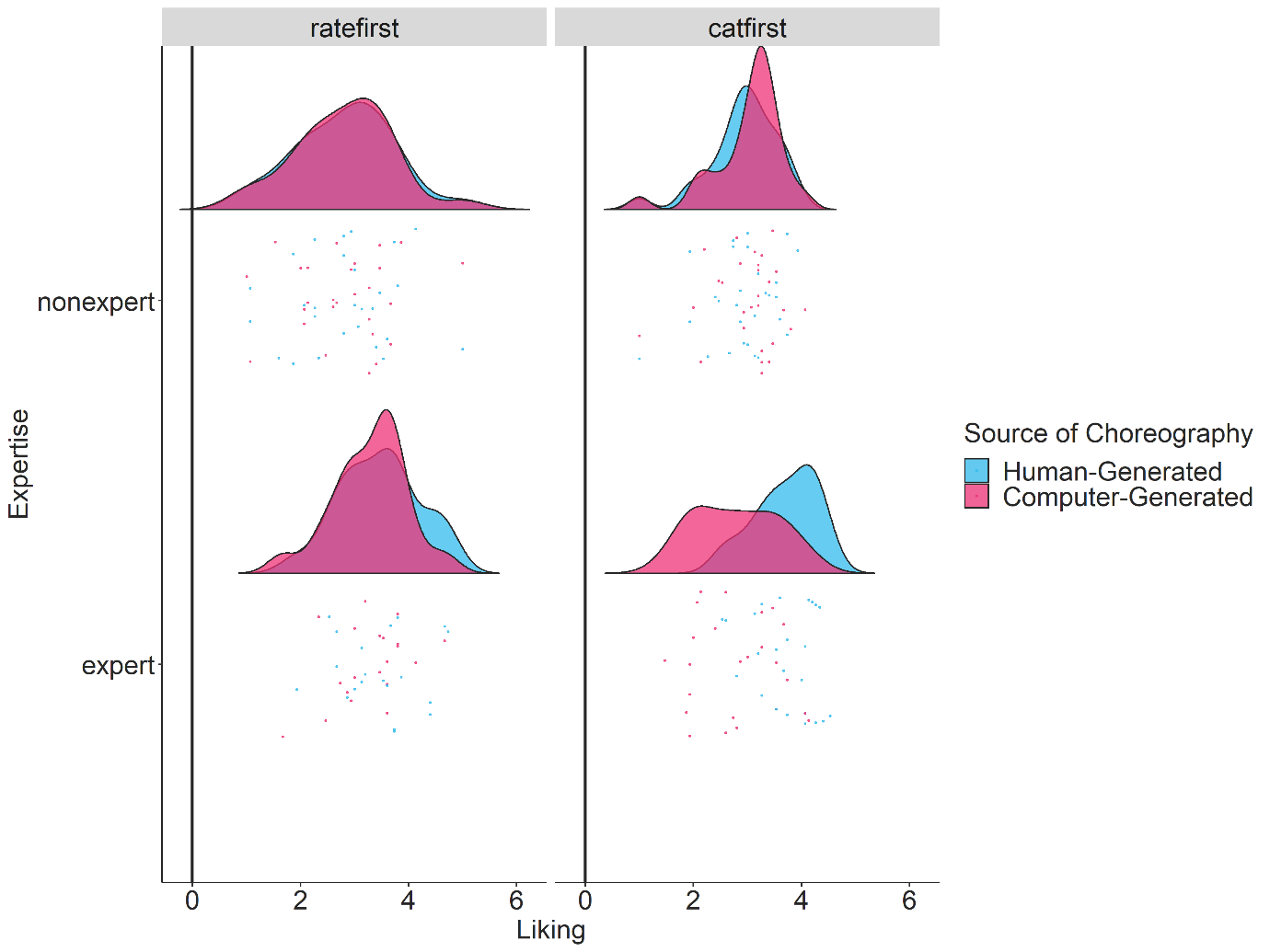


**Dance Expertise Questions**

On average, you see a dance performance once every:

Never (0) Year (1) 6 Months (2) 2 Months (3) Month (4) Week (5)

On average, you perform dance (on-stage/live/with a virtual audience) once every:

Never (0) Year (1) 6 Months (2) 2 Months (3) Month (4) Week (5)

On average, you read about dance theory once every:

Never (0) Year (1) 6 Months (2) 2 Months (3) Month (4) Week (5)

In the average week, how many hours do you spend dancing (as a hobby or practicing, not practising)

Never (0) Year (1) 6 Months (2) 2 Months (3) Month (4) Week (5)
